# Deletion of C3G in hepatocytes impairs full liver maturation and alters glucose homeostasis

**DOI:** 10.1038/s41419-025-08031-y

**Published:** 2025-10-07

**Authors:** Nerea Palao, Jaime Mancebo, Cristina Baquero, Minerva Iniesta-González, Mateo Cueto-Remacha, María Rodrigo-Faus, Alvaro Gutierrez-Uzquiza, Paloma Bragado, Ángel M. Cuesta, Aránzazu Sánchez, Carmen Guerrero, Almudena Porras

**Affiliations:** 1 Departamento de Bioquímica y Biología Molecular, Facultad de Farmacia, Universidad Complutense de Madrid, Madrid, Spain; 2 Instituto de Investigación Sanitaria del Hospital Clínico San Carlos (IdISSC), Madrid, Spain; 3 Centro de Investigación Biomédica en Red de Enfermedades Hepáticas y Digestivas (CIBEREHD-ISCIII), Madrid, Spain; 4 Instituto de Biología Molecular y Celular del Cáncer (IMBCC), Universidad de Salamanca-CSIC, Salamanca, Spain; 5 Instituto de Investigación Biomédica de Salamanca (IBSAL), Salamanca, Spain; 6 Departamento de Medicina, Universidad de Salamanca, Salamanca, Spain

**Keywords:** Biochemistry, Pathogenesis

## Abstract

C3G (RapGEF1) regulates the biology of liver hepatic progenitor cells and hepatocarcinoma cells, but its role in hepatocytes remained unknown. Therefore, we generated a mouse model lacking C3G in hepatocytes (C3GKO^Alb^), which showed liver damage as evidenced by increased fibrosis, liver macrophages and serum transaminases activity. Furthermore, impaired liver maturation was observed in C3GKO^Alb^ mice demonstrated by the low expression of hepatocyte specific proteins (i.e. HNF4α), but higher levels of Alpha-fetoprotein, and stemness markers (i.e. CD133). Glucose homeostasis was also altered in C3GKO^Alb^ mice, as well as insulin and glucagon effects on hepatocytes, which resulted in reduced serum glucose levels and an enhanced response to glucagon. In addition, the expression of several glycolytic and gluconeogenic enzymes, as well as the levels of the active form of Glycogen phosphorylase (PYGL), were upregulated in livers from C3GKO^Alb^ mice, being remarkable the increased Pyruvate kinase isoform 2 (PKM2) levels accompanied by higher serum lactate concentrations. An increased expression of the ketogenic enzyme 3-hydroxy 3-methylglutaryl-CoA (HMG) synthase (*Hmgcs2*) was also found in these livers in parallel to elevated blood levels of beta-hydroxy-butyrate. Moreover, the fasting response was enhanced in C3GKO^Alb^ mice as compared to wt animals. Hence, livers lacking C3G in hepatocytes showed a higher expression of gluconeogenic, lipogenic and ketogenic enzymes than livers from wt mice and enhanced ketogenesis. Mechanistically, data support a PTBP1-mediated upregulation of PKM2 expression in hepatocytes lacking C3G, which leads to enhanced glycolysis. Other metabolic alterations are likely due to the defective insulin signaling and the enhanced glucagon signaling through a cAMP-PKA-dependent mechanism. In summary, we have identified a novel role for C3G in the liver as a key mediator of hepatocyte differentiation and metabolic functions of hepatocytes. Hence, its absence leads to an immature phenotype and an altered response to insulin and glucagon, favoring glucagon actions.

## Introduction

The liver is an essential organ for numerous physiological processes such as glucose and lipid homeostasis, xenobiotic degradation and bile production. It is constituted by several cell types, including hepatocytes, biliary epithelial cells (cholangiocytes), hepatic stellate cells (HSCs), Kupffer cells and liver sinusoidal endothelial cells [1, 2]. Hepatocytes are the primary liver epithelial cell population. They derive from hepatoblasts, bipotential progenitor cells that give rise to both hepatocytes and cholangiocytes during liver development, which express markers of both lineages, including alpha-fetoprotein (AFP), albumin (ALB), hepatocyte nuclear factor-4α (HNF-4α) and cytokeratin-19 [3–5].

The liver function is highly regulated due to its role in metabolic homeostasis. Hepatic metabolism adapts to energy demands during development and in adult life [6]. At the end of gestation, glucose production is favored. After weaning, changes in the expression of liver metabolic enzymes facilitate glycolysis, fatty acid synthesis and oxidation, and ketogenesis [7]. Liver metabolism also adapts to different physio-pathological circumstances such as fasting, maintaining glucose homeostasis [8, 9]. During fasting, insulin decreases and glucagon increases, promoting glycogenolysis and gluconeogenesis [10]. In addition, fatty acids from adipose tissues are oxidized in liver mitochondria to generate energy or ketone bodies, which are exported to provide fuels for some extrahepatic tissues [11]. In contrast, when carbohydrates are abundant, they can be converted into fatty acids, and glycolysis and glycogenesis are activated [12].

The liver has a great regenerative capacity in response to damage. Upon short-term injury, liver fibrosis develops, contributing to tissue repair. HSCs are activated and transdifferentiate into myofibroblasts that produce collagen, which accumulates in the liver [13**–**15]. If liver damage becomes chronic, fibrosis can evolve to cirrhosis, eventually leading to liver cancer, with immune cells and platelets playing relevant roles [16**–**19].

C3G (Crk SH3-domain-binding guanine-nucleotide-releasing factor), encoded by the *RapGEF1* gene, is a guanine nucleotide exchange factor (GEF) for Rap1 [20], which can also act through GEF independent mechanisms [21**–**23]. C3G regulates several cellular processes, including proliferation, differentiation, cell death, migration [24**–**27], and glucose transport [28**–**30]. C3G is highly expressed in adult hepatic progenitor cells (HPCs) and neonatal hepatocytes, while low levels are found in adult hepatocytes [31]. C3G regulates HPC biology [32] with its down-regulation favoring stemness and migration. Interestingly, C3G expression is upregulated in hepatocellular carcinoma (HCC), promoting tumor growth [13, 31, 33].

Rap1a, the main C3G target as a GEF, regulates hepatocyte metabolism and glucose homeostasis. Specifically, Rap1a silencing promotes gluconeogenesis in hepatocytes [34].

Based on the functions of C3G in HPCs and Rap1a in hepatocytes, we hypothesized that C3G could regulate hepatocyte differentiation and functionality. Therefore, we generated a mouse model deficient in C3G in hepatocytes to define the physiological role of hepatocyte C3G during liver development and its function on metabolism.

## Materials and Methods

### Generation of a mouse model lacking C3G in hepatocytes

**Fig. 1:**
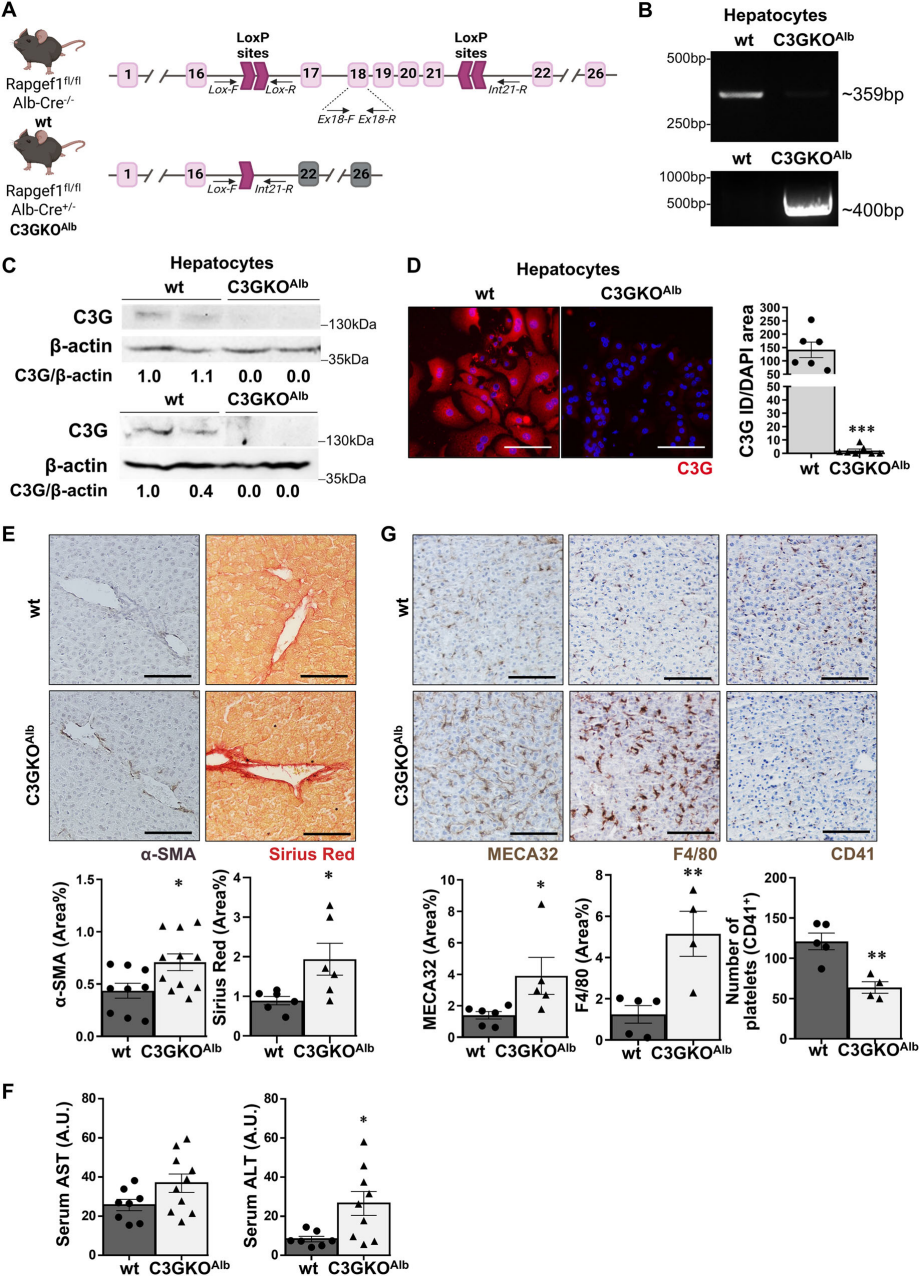
C3G deletion in hepatocytes induces liver damage. Mice lacking C3G in hepatocytes and their corresponding wt (*Rapgef*^*fl/fl*^*; AlbCre*^−/−^) littermates were generated, and their phenotype was analyzed. **A** Scheme showing that *Rapgef*^*fl/fl*^ mice with exons 17–21 flanked by loxP sites were crossed with *AlbCre*^+/−^ mice expressing Cre recombinase under the Albumin promoter to generate *Rapgef*^*fl/fl*^*; AlbCre*^+/−^ mice (C3GKO^Alb^) that show deletion of *Rapgef1* exons 17–21 in hepatocytes. **B** PCR analysis of hepatocyte genomic DNA using primers to detect floxed alleles (C3G-Ex18-f and C3G-Ex18-r) in wt mice and deletion of exons 17–21 (C3G-KO-LoxF and C3G-Int21R) in C3GKO^Alb^ mice. **C** Western blot analysis of C3G protein levels in hepatocytes from wt and C3GKO^Alb^ mice using two different C3G antibodies normalized with β-actin. **D** Immunofluorescence analysis of C3G protein levels in hepatocytes with a custom-made antibody. Nuclei were stained with DAPI. The histogram shows C3G versus DAPI quantification (integrated intensity). **E** Immunohistochemistry analysis of α-SMA (left) and Sirius Red staining of collagen (right) in liver sections from 1-month-old mice. Histograms in the lower panels show their quantification. **F** Histograms showing the activities of serum AST and ALT from 1-month-old mice. **G** Immunohistochemistry analysis of MECA32, F4/80, and CD41 staining in liver sections. Histograms in the lower panels show their quantification. *p ≤ 0.05, **p ≤ 0.01 and ***p ≤ 0.001 compared to wt mice (*n* = 4–12).

A hepatocyte-specific C3G knock-out mouse model (Rapgef1^flox/flox^; Albumin-Cre^+/−^, hereinafter C3GKO^Alb^) was generated by crossing Rapgef1^flox/flox^ mice [35] with Alb-Cre transgenic mice (B6.Cg-Speer6-ps1Tg(Alb-cre)21Mgn/J, JAX, Bar Harbor, ME, USA, strain 003574). Rapgef1^flox/flox^; Albumin-Cre^−/−^ mice (hereinafter wt) were used as controls (Fig. 1 and Supplementary Tables 1–3 and Supplementary Fig. 1).

### Fasting induction and glucose tolerance test (GTT)

To perform metabolic studies wt and C3GKO^Alb^ mice were fasted for different periods of time or fed ad libitum.

For the GTT, glucose was injected to 16 h-fasted mice and blood glucose was measured at different times using glucose strips (ACCU-CHEK Aviva, San Diego, CA, USA, 06453970).

All animal experiments were carried out in compliance with the European Community Council Directive (2010/63/EU) and following guidelines for animal research from Complutense University Ethical Committee, approved by Comunidad de Madrid (Spain) (PROEX 226.25-21, PROEX 198.3/22 and PROEX 176.0/24).

### Analysis of serum glucose, transaminases, insulin, glucagon, beta-hydroxy-butyrate and lactate

Serum glucose levels were quantified using Glucose-TR kit (Spinreact, Sant Esteve de Bas, Spain, 1001190); and serum aspartate aminotransferase (AST) and alanine aminotransferase (ALT) activities using specific kits (Spinreact, 41273 and 1001172, respectively).

Serum insulin and glucagon levels were measured using ELISA or EIA kits (Thermo Fisher Scientific, Waltham, MA, USA, EMINS and Sigma, Burlington, MA, USA, RAB0202, respectively).

Serum beta-hydroxy-butyrate was determined using a Kit (Cliniscience, Nanterre, France, MA-BHB-1).

Lactate was determined using lactate dehydrogenase (Roche, 10127876001, Basel, Switzerland) for conversion of lactate to pyruvate measuring NADH generation.

### Immunohistochemistry (IHC), collagen and glycogen analysis in liver sections

Paraffin embedded liver sections were dewaxed, permeabilized, blocked and incubated with antibodies against: α-SMA, F4/80, CD41, and MECA32. After washing, sections were incubated with biotinylated secondary antibodies, avidin/biotin (1:1), revealed with 3,3′-diaminobenzidine, hematoxylin counterstained and mounted using DPX. Images were taken using an Eclipse TE300 Nikon microscope coupled to a Digital Sight DS-U2 camera (20X) or a Leica DMC 4500 microscope.

Sirius Red was used to stain liver collagen.

Periodic acid Schiff staining was used to detect liver glycogen.

### Isolation, culture and stimulation of adult hepatocytes

Hepatocytes were isolated from the liver of 2–3-month-old mice as previously described [36, 37] through digestion with collagenase IV and purification using a Percoll density gradient. Hepatocytes were seeded on collagen I pre-coated plates. The experiments were performed within the next 24 h.

### Immunofluorescence analysis in the liver and adult hepatocytes

Liver sections were prepared as described for IHC. Hepatocytes were fixed and permeabilized. After washing and blocking, sections or cells were incubated with primary antibodies against ALB, C3G, cleaved-Caspase 3, CD133, HNF4-α, E-cadherin and PKM2. After washing, they were incubated with the secondary antibody and 4′,6-diamidino-2-phenylindole (DAPI), washed, and mounted using Prolong Gold Antifade Reagent.

Images were taken in either a Nikon Eclipse TE300 epifluorescence microscope or a Leica DMC 4500 microscope.

### RNA isolation and reverse transcriptase polymerase chain reaction (RT-qPCR)

Total RNA isolation and RT-qPCR analysis were performed as previously described [31]. cDNA was amplified using specific primers normalizing with *Gusb* gene (Supplementary Table 4).

### Western blot analysis

Western blot analysis was carried out as previously described [31]. Membranes were probed with specific primary antibodies (Supplementary Table 5) and β-Actin or α-Tubulin to normalize.

### Statistical analysis

Data were represented as the mean values ± S.E.M. (standard error of the mean) of, at least, three independent experiments. Unpaired Student’s *t* test was used to compare two experimental groups. One-way or two-way analysis of variance was used to compare more than two groups, followed by a multiple comparison Bonferroni test. Statistical significance was considered when *p* value ≤0.05.

Materials and methods are further described in Supplementary Information.

## Results

### C3G deletion in hepatocytes induces liver damage and hepatocyte immaturity

We have generated and characterized a mouse model lacking C3G in hepatocytes (C3GKO^Alb^) to determine its role in liver development and function. In this mouse, exons 17–21 were deleted in Alb-Cre expressing cells (Fig. 1A), leading to C3G knockout as demonstrated by PCR analysis (Fig. 1B), western blot (Fig. 1C) and immunofluorescence analysis (Fig. 1D) in isolated adult hepatocytes. The levels of other Rap1GEFs, Epac1 and Epac2, were similar in livers from C3GKO^Alb^ and wt mice (Supplementary Fig. 2A), while Rap1 protein levels increased in C3GKO^Alb^ hepatocytes (Supplementary Fig. 2B), likely to compensate for C3G loss.

Although general liver morphology was normal (Supplementary Fig. 2C) in C3GKO^Alb^ compared to wt mice, increased levels of α-SMA and collagen (Fig. 1E) and higher serum AST/ALT activities (Fig. 1F) were detected, suggesting the presence of liver damage. Supporting this, more vascularization (MECA32 staining), macrophages (F4/80 positive cells), and less platelets (CD41 staining) were also found in livers from C3GKO^Alb^ mice (Fig. 1G). Apoptosis was also increased in C3GKO^Alb^ hepatocytes as evidenced by cleaved-Caspase 3 data (Supplementary Fig. 2D).

**Fig. 2:**
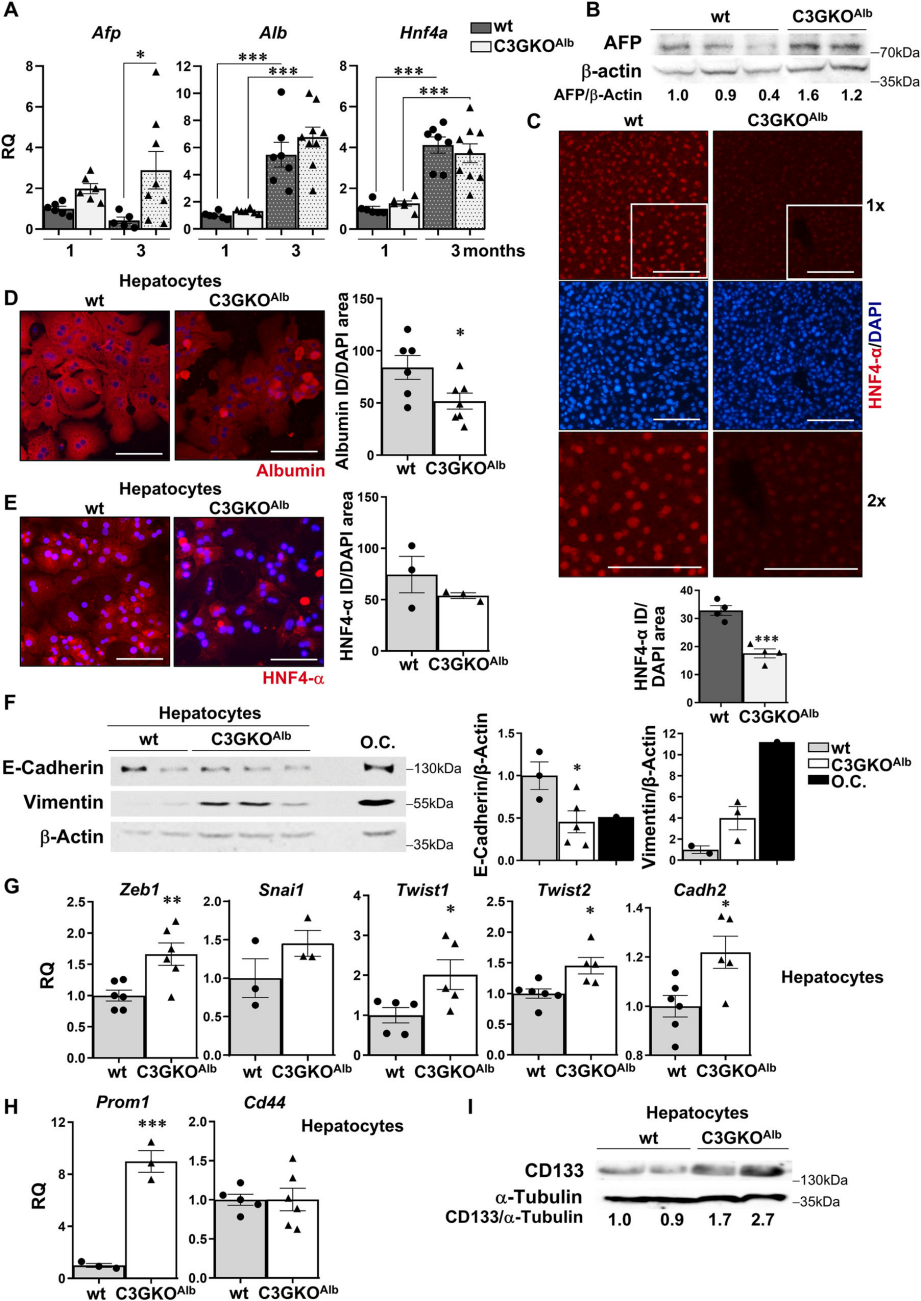
C3G deficiency in hepatocytes impairs full hepatocyte and liver maturation. **A** RT-qPCR analysis of *Afp,* *Alb*, and *Hnf4a* mRNA expression in the liver of 1- and 3-month-old C3GKO^Alb^ and wt mice expressed as RQ (relative quantification). **B** Western blot analysis of AFP protein levels in the liver of 1-month-old C3GKO^Alb^ and wt mice normalized with β-actin. **C** Immunofluorescence analysis of HNF4α protein levels in liver sections from 1-month-old C3GKO^Alb^ and wt mice. ×1 images and zoom-in areas of the ×1 images (white square) are shown (×2). Nuclei were stained with DAPI. The histogram shows the quantification of HNF4α versus DAPI-positive area (%). **D** Immunofluorescence analysis of Albumin protein levels in hepatocytes from C3GKO^Alb^ and wt mice. Nuclei were stained with DAPI. The histogram shows the quantification of Albumin versus DAPI-positive area (expressed as %). **E** Immunofluorescence analysis of HNF4α protein levels in hepatocytes from C3GKO^Alb^ and wt mice. Nuclei were stained with DAPI. The histogram shows the quantification of HNF4α versus DAPI-positive area (in %). **F** Western blot analysis of E-cadherin and Vimentin protein levels in hepatocytes from C3GKO^Alb^ and wt mice normalized with β-actin. O.C. oval cells. **G** RT-qPCR analysis of *Zeb1*, *Snai1*, *Twist1*, *Twist2,* and *Cadh2* mRNA expression in hepatocytes from C3GKO^Alb^ and wt mice. **H** RT-qPCR analysis of *Prom1* and *Cd44* mRNA expression in hepatocytes from C3GKO^Alb^ and wt mice. **I** Western blot analysis of CD33 protein levels in hepatocytes from C3GKO^Alb^ and wt mice normalized with β-Actin. *p ≤ 0.05, **p ≤ 0.01, ***p ≤ 0.001 compared to wt mice (*n* = 2–6).

The expression of differentiation markers was also altered. Increased mRNA and protein levels of AFP, marker of hepatocyte immaturity, were found in livers of C3GKO^Alb^ compared to wt mice (Fig. 2A, B). Although *Alb* and *Hnf4a* mRNA levels were similar in wt and C3GKO^Alb^ livers (Fig. 2A), their protein levels were lower in livers and/or hepatocytes from C3GKO^Alb^ than from wt mice (Fig. 2C–E).

In addition, the levels of the epithelial marker E-cadherin decreased in C3GKO^Alb^ compared to wt hepatocytes, while those of Vimentin (mesenchymal marker) increased (Fig. 2F and Supplementary Fig. 3A). In agreement with this, the expression of the mRNAs *Zeb1*, *Snai1*, *Twist1*, and *Twist2*, encoding transcription factors involved in epithelial–mesenchymal-transition induction, and *Cdh2* mRNA (encoding N-cadherin) increased in C3GKO^Alb^ compared with wt hepatocytes (Fig. 2G). All this is indicative of a less epithelial phenotype of C3GKO^Alb^ hepatocytes and therefore, lower maturity. Additionally, the expression of the stemness marker *Prom1* mRNA (encoding CD133) (Fig. 2H) and CD133 protein was upregulated in C3GKO^Alb^ hepatocytes (Fig. 2I and Supplementary Fig. 3B), suggesting incomplete differentiation towards hepatocytes, although *Cd44* mRNA levels were similar to wt values (Fig. 2H).

All this supports that C3G would be essential for full hepatocyte differentiation.

### The absence of C3G in hepatocytes reduces blood glucose levels, altering glucose metabolism

**Fig. 3:**
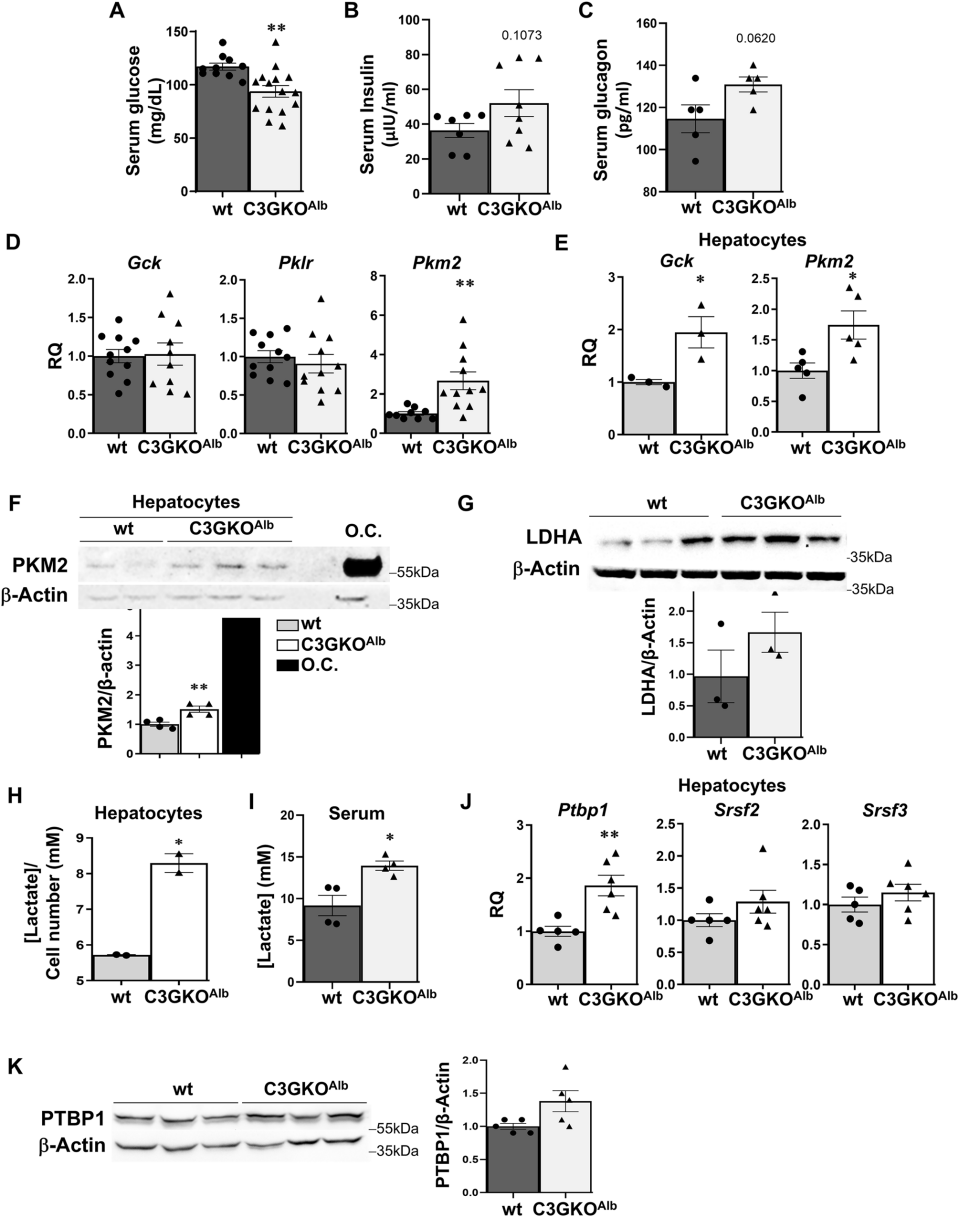
Glucose homeostasis is altered in mice lacking C3G in hepatocytes. Serum levels of glucose (**A**) insulin (**B**) and glucagon (**C**) in 1-month-old C3GKO^Alb^ and wt mice. **D** RT-qPCR analysis of *Gck, Pklr*, and *Pkm2* mRNA expression in the liver of 1-month-old C3GKO^Alb^ and wt mice expressed as RQ (relative quantification). **E** RT-qPCR analysis of *Gck* and *Pkm2* mRNA expression in hepatocytes from C3GKO^Alb^ and wt mice expressed as RQ. **F** Western blot analysis of PKM2 protein levels in hepatocytes from C3GKO^Alb^ and wt mice normalized with β-Actin. O.C. oval cells. **G** Western blot analysis of LDHA protein levels in livers from C3GKO^Alb^ and wt mice normalized with β-Actin and referred to wt values. Histogram shows the quantification of LDHA versus β-Actin. **H** Lactate concentration in the culture medium of hepatocytes from C3GKO^Alb^ and wt mice. **I** Serum lactate concentration in 1-month-old C3GKO^Alb^ and wt mice. **J** RT-qPCR analysis of *Ptbp1, Srsf2*, and *Srsf3* mRNA expression in hepatocytes from C3GKO^Alb^ and wt mice expressed as RQ. **K** Western blot analysis of PTBP1 protein levels in livers from C3GKO^Alb^ and wt mice normalized with β-Actin. *p ≤ 0.05, **p ≤ 0.01 compared to wt mice (*n* = 2–10).

Considering the liver immaturity of C3GKO^Alb^ mice and the relevance of the liver in the control of glucose homeostasis, we evaluated blood glucose levels in 1-month-old mice. Basal serum glucose levels were lower in C3GKO^Alb^ mice (Fig. 3A), while insulin levels tended to be upregulated (Fig. 3B). Curiously, serum glucagon levels were also increased (Fig. 3C). Hence, we analyzed the expression of enzymes involved in glucose metabolism (Supplementary Fig. 4).

First, glycolytic enzymes levels were determined. Glucokinase (*Gck*) and Pyruvate kinase L (*Pklr*) mRNA expression was similar in livers from C3GKO^Alb^ and wt mice (Fig. 3D), although *Gck* mRNA levels increased in C3GKO^Alb^ hepatocytes (Fig. 3E). Notably, the expression of PKM2 isoform (mRNA and protein) was upregulated in both livers and adult hepatocytes from C3GKO^Alb^ compared to wt mice (Fig. 3D–F), PKM2 being present in cytosol and nuclei, especially in C3GKO^Alb^ hepatocytes (Supplementary Fig. 5A). All this suggests that glycolysis could be enhanced in the liver when C3G is deleted in hepatocytes, which is supported by increased lactate dehydrogenase A (LDHA) levels and lactate generation by C3GKO^Alb^ hepatocytes (Fig. 3H), together with a higher serum lactate concentration (Fig. 3I).

The increased *Pkm2* mRNA levels in the liver and hepatocytes from C3GKO^Alb^ *vs* wt mice suggests that *Pkm* splicing is shifted towards *Pkm2* isoform expression. Since C3G can regulate the expression of different splicing factors such as SRF2 in muscle cells [38], and PKM2 splicing is regulated by PTBP1 and SRSF3 splicing factors, we analyzed *Ptbp1*, *Srsf2*, and *Srsf3* mRNA levels in liver and hepatocytes. Although no differences between genotypes were detected in the liver (Supplementary Fig. 5B), *Ptbp1* mRNA levels were upregulated in C3GKO^Alb^ hepatocytes (Fig. 3G) and PTBP1 protein levels tended to increase in livers from C3GKO^Alb^ mice (Fig. 3H), suggesting its involvement in the increased PKM2 expression. Moreover, PKM2 and PTBP1 protein levels were also upregulated in the C3G-silenced HLE HCC cell line (Supplementary Fig. 5C, D). More importantly, upon PTBP1 silencing PKM2 protein expression was reduced by 40–50% in C3G-silenced HLE cells and 10% in non-silenced cells (Supplementary Fig. 5D). Additionally, *PKM1* mRNA expression tended to increase, while *PKM2* mRNA levels decreased in C3G-silenced HLE cells upon PTBP1 silencing (Supplementary Fig. 5E), increasing *PKM1*/*PKM2* ratio. Therefore, C3G likely regulates *PKM* splicing through PTBP1.

In view of the lower blood glucose levels found in C3GKO^Alb^ compared to wt mice under feeding conditions, liver glycogen was measured. Less glycogen was present in C3GKO^Alb^ than in wt livers (Supplementary Fig. 6A). Although *Pygl* mRNA (encoding glycogen phosphorylase) levels were lower in livers from C3GKO^Alb^, and no significant changes were observed in *Gys* mRNA (encoding glycogen synthase (GS)) expression (Supplementary Fig. 6B). However, total PYGL and phospho-PYGL (active form) levels increased (Supplementary Fig. 6C). This supports enhanced glycogenolysis. Moreover, GS and P-GS (inactive form) protein levels were similar in livers from wt and C3GKO^Alb^ mice, ruling out differences in glycogen synthesis.

**Fig. 4:**
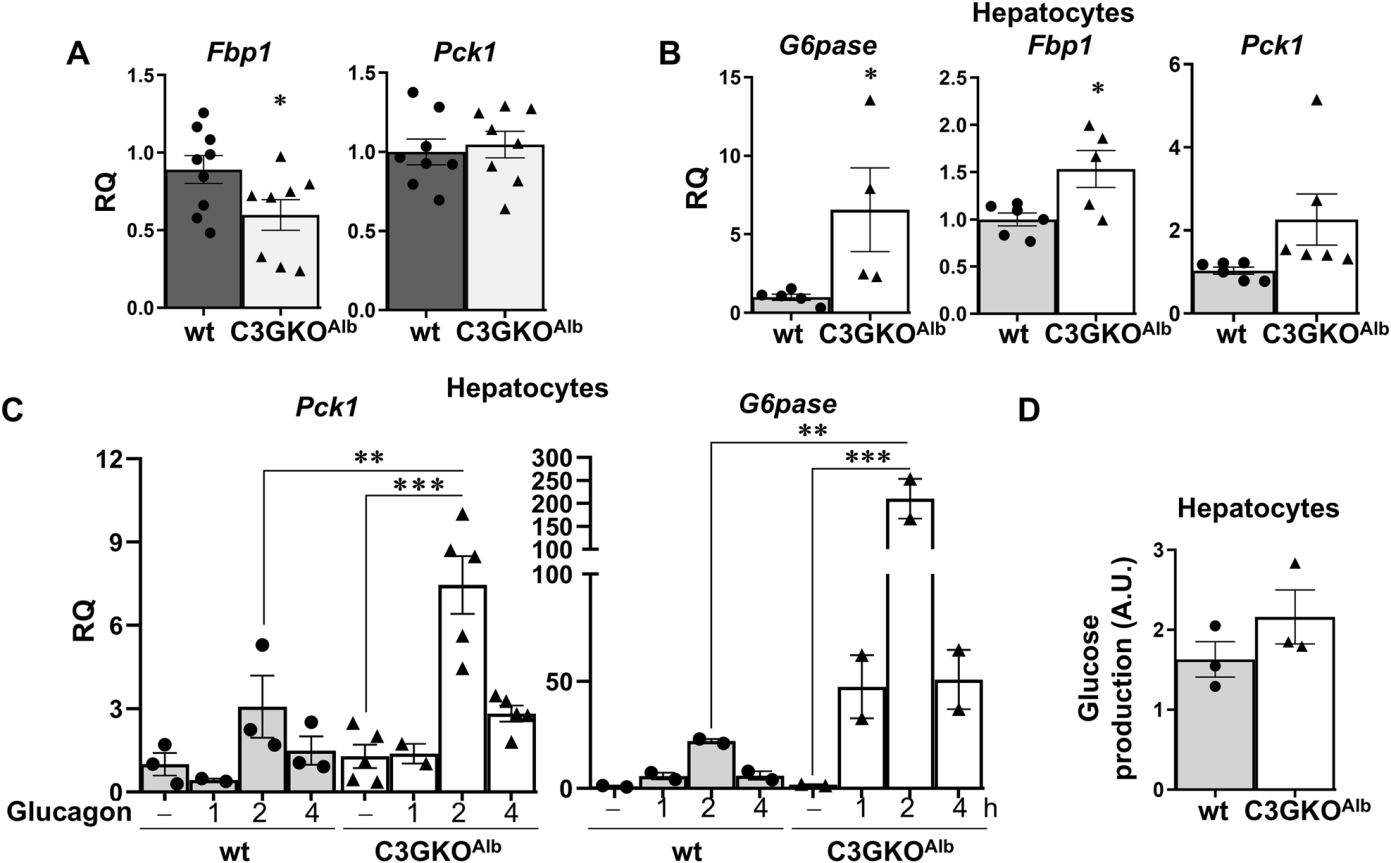
C3G deletion in hepatocytes increases the expression of gluconeogenic enzymes, enhancing the glucagon effect. **A** RT-qPCR analysis of *Fbp1* and *Pck1* mRNA expression in the liver of 1-month-old C3GKO^Alb^ and wt mice. **B** RT-qPCR analysis of *G6pase, Fbp1*, and *Pck1* mRNA expression in hepatocytes from C3GKO^Alb^ and wt mice. **C** RT-qPCR analysis of *G6pase* and *Pck1* mRNA expression in hepatocytes from C3GKO^Alb^ and wt mice stimulated with glucagon for 1, 2, and 4 h, or maintained untreated. **D** Glucose production by hepatocytes from C3GKO^Alb^ and wt mice stimulated with glucagon for 4 h expressed in arbitrary units (A.U.). *p ≤ 0.05, **p ≤ 0.01, ***p ≤ 0.001 compared to wt mice (*n* = 2–8).

**Fig. 5:**
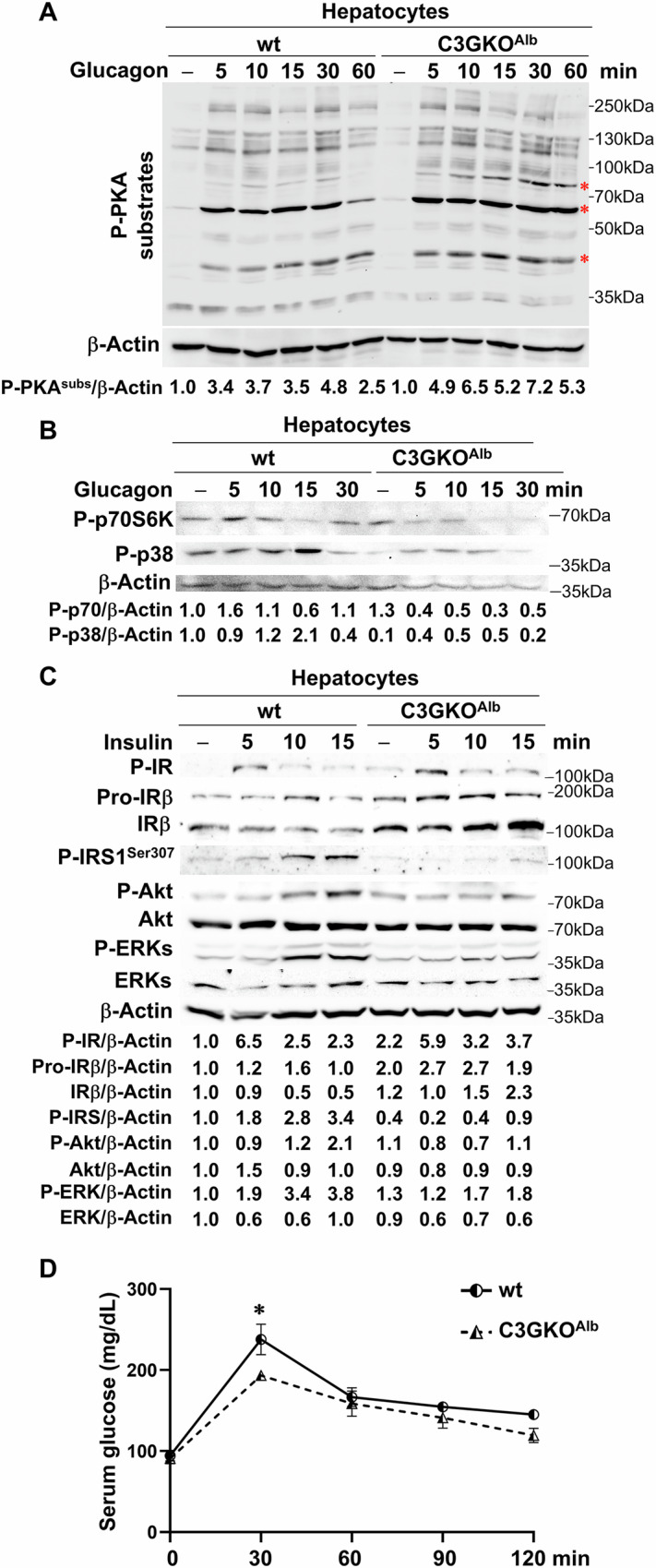
C3G deletion in hepatocytes increases glucagon response, decreases insulin signaling, and improves glucose tolerance. **A** Western blot analysis of P-PKA substrates in hepatocytes from C3GKO^Alb^ and wt mice stimulated with glucagon for 5, 10, 15, 30, and 60 min, or maintained untreated normalized with β-Actin. **B** Western blot analysis of P-p38 and P-p70S6K (P(Thr385)-p70S6K) in hepatocytes from C3GKO^Alb^ and wt mice stimulated with glucagon for 5, 10, 15, and 30 min, or maintained untreated normalized with β-Actin. **C** Western blot analysis of P^-^IR^Tyr1345^, Pro-IRβ, IRβ, P-IRS1^Ser307^, P-Akt, Akt, P-ERKs, and ERKs in hepatocytes from C3GKO^Alb^ and wt mice stimulated with insulin for 5, 10, and 15 min, or maintained untreated normalized with β-Actin. **D** Glucose tolerant test (GTT). Glucose was administered to 16 h-fasted mice, and blood glucose was measured at different time points. *p ≤ 0.05 compared to wt mice (*n* = 2–6).

We also analyzed the expression of gluconeogenic enzymes. A lower expression of *Fbp1* mRNA (encoding fructose-1,6-biphosphatase 1) was observed in livers from C3GKO^Alb^ compared to wt mice, while *Pck1* (encoding PEPCK) expression was similar (Fig. 4A). However, *G6pase, Fbp1*, and *Pck1* mRNA levels were increased in C3GKO^Alb^ compared to wt hepatocytes (Fig. 4B). Moreover, glucagon-induced *G6pase* and *Pck1* mRNA expression was greater in C3GKO^Alb^ hepatocytes (Fig. 4C), resulting in more glucose production (Fig. 4D). This indicates that hepatocytes lacking C3G have higher basal levels of gluconeogenic enzymes, which further increase upon glucagon treatment. In agreement with this, cAMP-PKA-dependent glucagon signaling was enhanced in C3GKO^Alb^ hepatocytes, increasing the phosphorylation levels of several PKA substrates (Fig. 5A), while p38MAPK and p70S6K activation was impaired (Fig. 5B).

C3GKO^Alb^ hepatocytes also showed a defective response to insulin in terms of Akt and ERKs activation (phosphorylated forms) (Fig. 5C). Although insulin receptor (IR) tyrosine phosphorylation was not altered and IR total levels increased, the feedback inhibition of insulin signaling through IRS1 phosphorylation in Ser307 was highly reduced in C3GKO^Alb^ hepatocytes (Fig. 5C). We also analyzed the mRNA levels of insulin and glucagon receptors. A tendency to increase *Insr* was found in livers from C3GKO^Alb^ compared to wt mice, while there were no changes in *Gcgr* levels (Supplementary Fig. 7).

Considering glucose metabolism alterations upon deletion of C3G in hepatocytes and the reduced basal blood glucose levels, a GTT assay was performed detecting a significant lower serum glucose concentration in C3GKO^Alb^ mice 30 min after glucose infusion (Fig. 5D). This suggests quicker serum glucose clearance in C3GKO^Alb^ mice.

### C3G deletion in hepatocytes increases ketogenesis

**Fig. 6:**
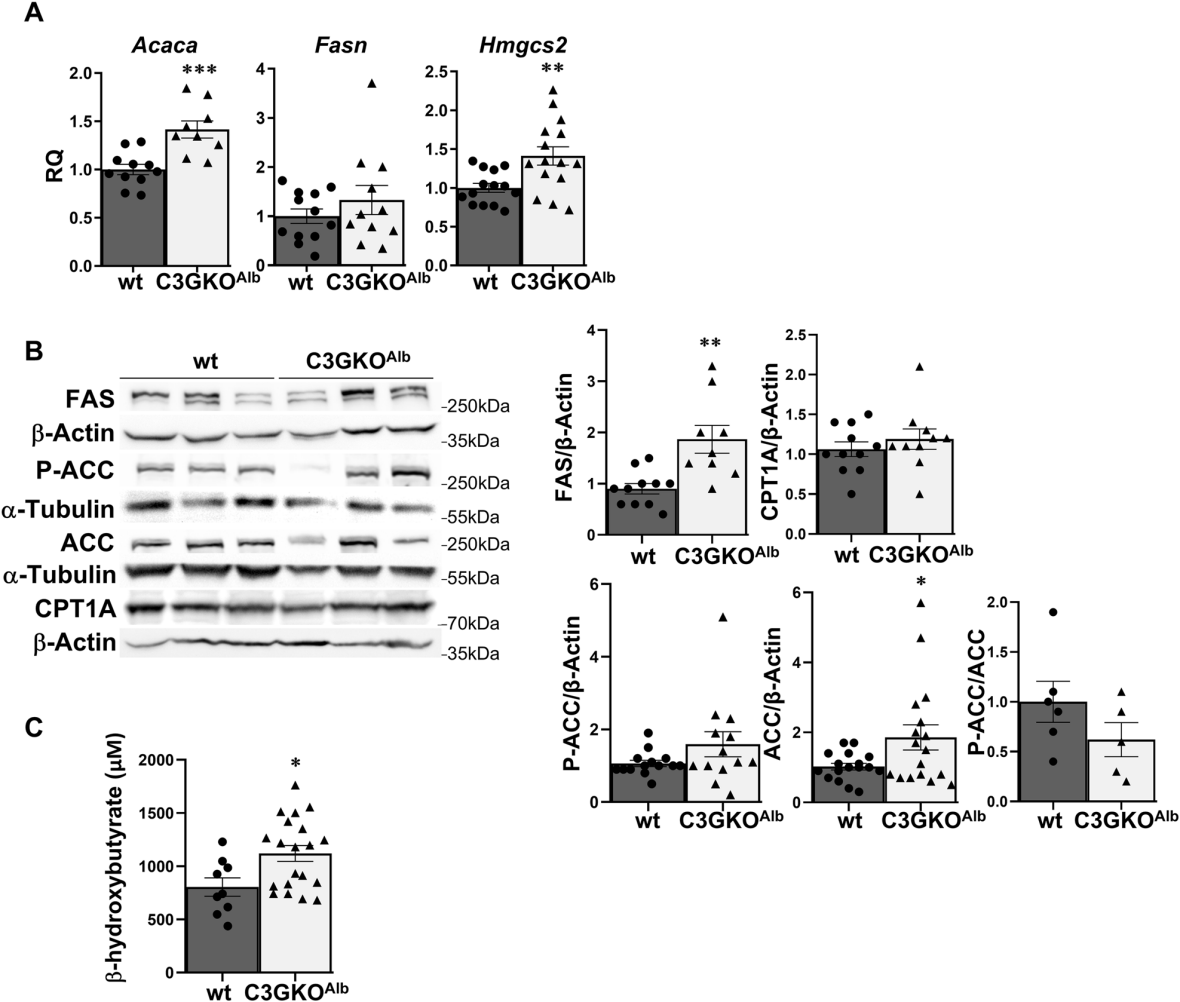
Impact of C3G deficiency in hepatocytes on the expression of enzymes involved in fatty acid metabolism and ketogenesis. **A** RT-qPCR analysis of *Acaca, Fasn*, and *Hmgcs2* mRNA expression in the liver of 1-month-old C3GKO^Alb^ and wt mice. **B** Western blot analysis of FAS, P-ACC, ACC, and CPT1A protein levels in livers from C3GKO^Alb^ and wt mice normalized with β-actin or α-Tubulin (left panel). Histograms show the quantification of different western blots (right panel). **C** Histogram showing serum levels of beta-hydroxy-butyrate. *p ≤ 0.05, **p ≤ 0.01, ***p ≤ 0.001 compared to wt mice (*n* = 2–15).

C3G deletion in hepatocytes alters liver glucose metabolism, therefore, fatty acid metabolism could be also affected. Results showed that in livers and hepatocytes from C3GKO^Alb^ mice compared to wt animals, mRNA levels of the lipogenic enzyme *Acaca* (encoding AcetylCoA carboxylase 1) increased, but not *Fasn* (encoding Fatty acid synthase) (Fig. 6A and Supplementary Fig. 8A). FAS and ACC protein levels were higher in the livers and hepatocytes from C3GKO^Alb^ mice compared to wt animals, and P-ACC/ACC ratio decreased (Fig. 6B and Supplementary Fig. 8B, C), suggesting enhanced lipogenesis. On the other hand, CPT1A levels were similar (Fig. 6B and Supplementary Fig. 8C), while the expression of *Hmgcs2* mRNA, encoding the ketogenic enzyme HMGCS2 (3-Hydroxy-3-Methylglutaryl-Coenzyme A Synthase 2), was upregulated in livers from C3GKO^Alb^ mice (Fig. 6A). In agreement with this, serum levels of beta-hydroxy-butyrate were higher in C3GKO^Alb^ mice (Fig. 6C).

According to these results, glucose could be diverted to fatty acid synthesis and then, fatty acids could be used to generate ketone bodies in C3GKO^Alb^ mice.

### Impact of hepatocyte C3G deletion on fasting response

**Fig. 7:**
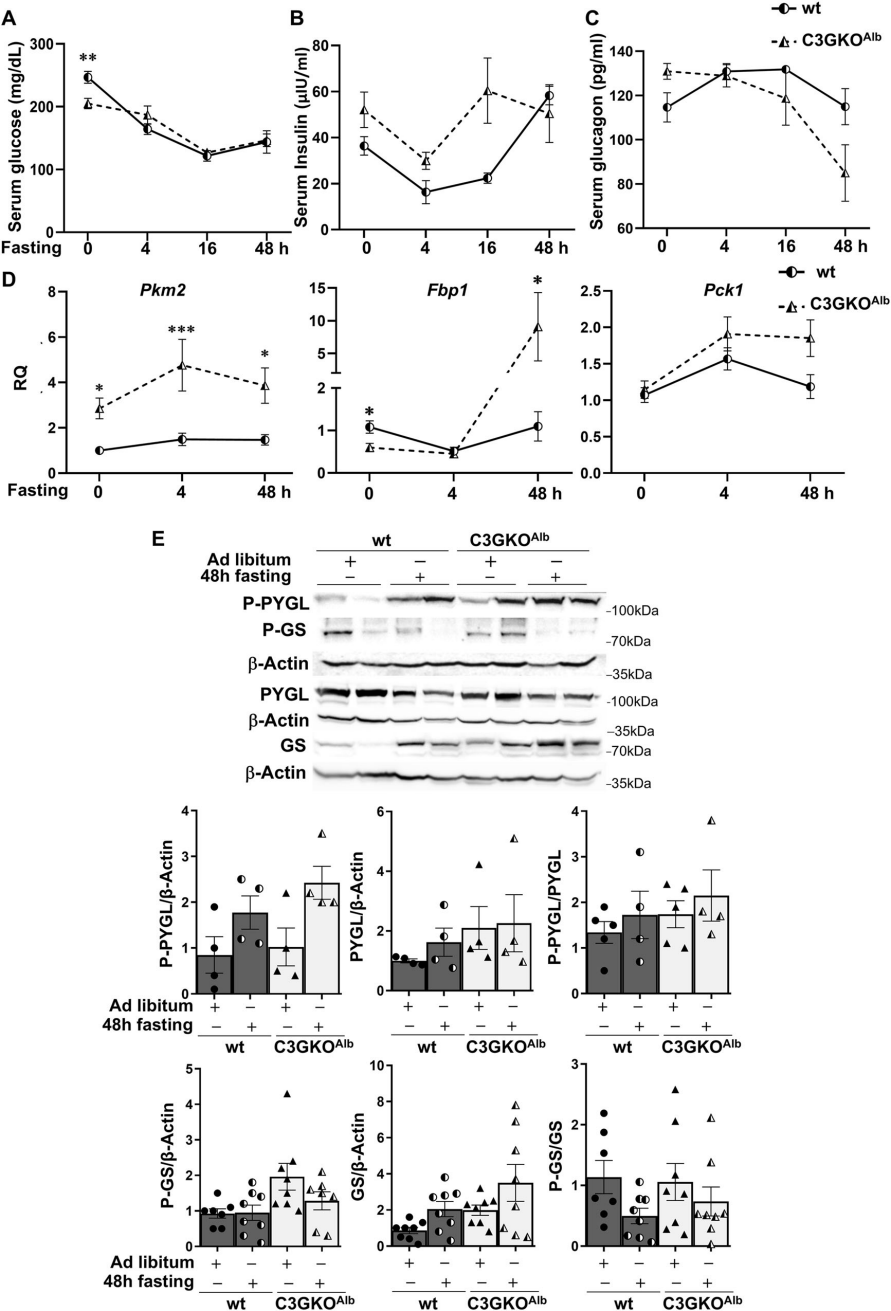
Mice lacking C3G in hepatocytes show alterations in glucose metabolism in response to fasting. Serum levels of glucose (**A**) insulin (**B**) and glucagon (**C**) in 1-month-old C3GKO^Alb^ and wt mice after fasting for 4, 16, and 48 h. **D** RT-qPCR analysis of *Pkm2*, *Fbp1*, and *Pck1* mRNA expression in the liver of 1-month-old C3GKO^Alb^ and wt mice after fasting for 4 and 48 h. **E** Western blot analysis of PYGL, P-PYGL, GS, and P-GS protein levels in livers from C3GKO^Alb^ and wt mice after fasting for 4 and 48 h normalized with β-Actin. Histograms show the quantification of different western blots. *p ≤ 0.05, **p ≤ 0.01, ***p ≤ 0.001 compared to wt mice (*n* = 4–6).

Based on the above-described liver metabolic alterations and the changes in serum glucose, lactate and beta-hydroxy-butyrate in ad libitum-fed C3GKO^Alb^ mice, we analyzed the fasting response. After 4 h of fasting, serum glucose levels decreased in wt mice, but not in C3GKO^Alb^ mice (Fig. 7A), while between 4 and 16 h of fasting, a similar sharp decrease was detected in both genotypes, with no further reduction after 48 h. On the other hand, although serum insulin levels tended to be upregulated in C3GKO^Alb^ mice, they decreased in both genotypes after 4 h of fasting, increasing afterwards (Fig. 7B). Curiously, serum glucagon levels were higher in fed C3GKO^Alb^, but decreased after 16 h of fasting, while in wt mice they increased after 4 h of fasting, recovering basal levels after 48 h (Fig. 7C).

During fasting, to maintain glucose homeostasis, liver glycogenolysis, gluconeogenesis and ketogenesis increase, decreasing glycolysis and glycogenesis [8, 39]. Hence, we evaluated the expression of enzymes from these metabolic processes. Curiously, the higher basal liver expression of *Pkm2* mRNA in C3GKO^Alb^ mice was further increased after 4 h of fasting, remaining high up to 48 h (Fig. 7D). In contrast, *Pkm2* mRNA expression remained low in wt livers during fasting.

Concerning gluconeogenic enzymes, both *Fbp1* and *Pck1* mRNA levels increased after 48 h of fasting in livers from C3GKO^Alb^ and wt mice, showing a higher expression in C3GKO^Alb^ mice (Fig. 7D). This suggests an enhancement of gluconeogenesis in livers from C3GKO^Alb^ mice during fasting.

Regarding glycogen metabolism, P-PYGL and P-PYGL/PYGL levels tended to increase more after 48 h of fasting in livers from C3GKO^Alb^, remaining higher than in wt mice (Fig. 7E). On the other hand, P-GS decreased after 48 h fasting in both wt and C3GKO^Alb^ mice, although P-GS and P-GS/GS levels tended to be higher in livers from C3GKO^Alb^ mice (Fig. 7E).

**Fig. 8:**
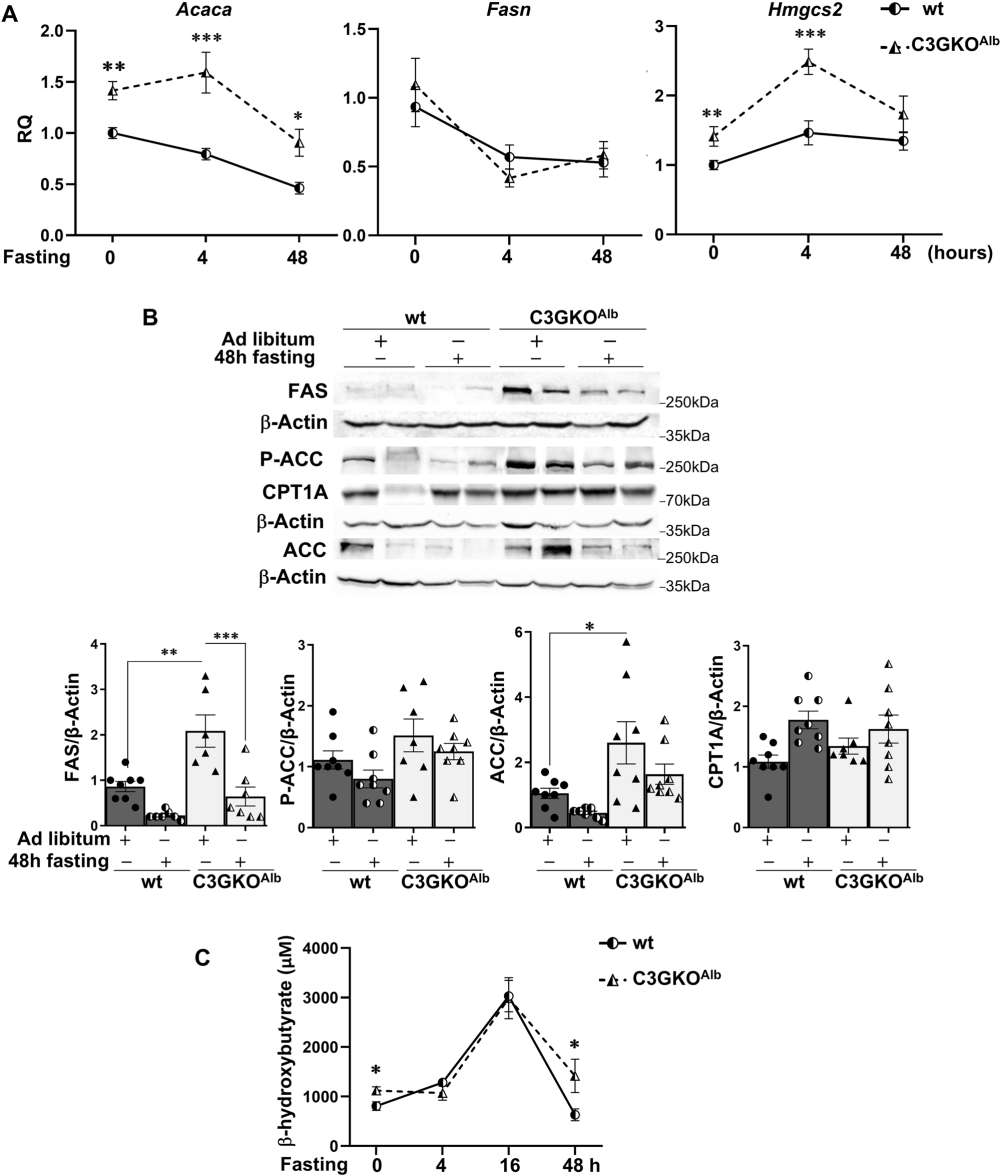
Altered expression of lipogenic and Ketogenic enzymes in fasted mice lacking C3G in hepatocytes. **A** RT-qPCR analysis of *Acaca, Fasn*, and *Hmgcs2* mRNA expression in the liver of 1-month-old C3GKO^Alb^ and wt mice after fasting for 4 and 48 h. **B** Western blot analysis of FAS, P-ACC, ACC, and CPT1A protein levels in livers from C3GKO^Alb^ and wt mice after fasting for 4 and 48 h normalized with β-Actin. Histograms show the quantification of different western blots. **C** The graphic shows serum levels of beta-hydroxy-butyrate in 1-month-old C3GKO^Alb^ and wt mice at different fasting times (4, 16, and 48 h). *p ≤ 0.05, **p ≤ 0.01, ***p ≤ 0.001 compared to wt mice (*n* = 4–8).

The expression of enzymes involved in fatty acid metabolism and ketogenesis also showed differences between wt and C3GKO^Alb^ mice during fasting. *Acaca* mRNA levels were significantly higher in livers from C3GKO^Alb^ mice under feeding and fasting conditions (Fig. 8A). Unlike this, *Fasn* mRNA levels were strongly reduced after 4 h of fasting in both wt and C3GKO^Alb^ mice with no additional changes afterwards. On the other hand, ACC, P-ACC and FAS protein levels were higher in livers from fed and 48 h-fasted C3GKO^Alb^ mice, although they decreased 48 h after fasting (Fig. 8B). In contrast, CPT1A tended to increase 48 h after fasting in both genotypes. Moreover, *Hmgcs2* mRNA expression increased after 4 h fasting in livers from both C3GKO^Alb^ and wt mice, decreasing afterwards, reaching significantly higher levels in C3GKO^Alb^ mice (Fig. 8A). In agreement with this, C3GKO^Alb^ mice had higher serum β-hydroxy-butyrate levels after 48 h of fasting (Fig. 8C).

All this suggests that, under both feeding and fasting conditions, livers from C3GKO^Alb^ mice express higher levels of enzymes from fatty acid synthesis and ketogenesis with ketogenesis being enhanced upon fasting in C3GKO^Alb^ mice.

## Discussion

Our previous work evidenced that C3G is highly expressed in oval cells (HPCs) and neonatal hepatocytes [33] and regulates oval cell biology [32]. This suggests its potential involvement in hepatocyte differentiation, which is demonstrated in this work using a novel mouse model of C3G deletion in hepatocytes generated in our laboratory. Both liver and hepatocytes from these mice showed increased expression of AFP and the stemness marker CD133, while HNF4α and ALB levels were reduced. This agrees with the enhanced expression of stemness markers in C3G-silenced oval cells [32] and the impaired lineage commitment of C3G knock-out in mouse embryonic stem cells [40]. These data are also in line with the function of C3G promoting other differentiation processes such as those leading to megakaryocytes [41] and muscle cells [42].

C3G deletion in hepatocytes also alters liver metabolic function and induces liver damage, demonstrated by enhanced fibrosis, inflammation (macrophage accumulation) and serum AST/ALT activity under steady-state conditions. Among the alterations found, it is noticeable the increased expression of gluconeogenic (*G6pc* and *Pck1*) enzymes in fasted C3GKO^Alb^ mice compared to wt mice and the enhanced response to glucagon of C3GKO^Alb^ hepatocytes increasing *G6pc* and *Pck1* mRNA levels. This resembles the behavior of Rap1a-KO (or Epac2-silenced) hepatocytes compared to wt hepatocytes [34]. Therefore, C3G, in collaboration with Epac2, would regulate liver metabolism, at least partially, through Rap1 [34]. Additional reports also point to Epac2/Rap1 as mediators of glucagon metabolic [43, 44] and non-metabolic effects on the liver and hepatocytes [45, 46], and now, we can add C3G. In this line, the decrease in p38 MAPK activation found in C3G deficient hepatocytes (Fig. 5B) resembles the effect of Epac2 silencing [43], which impairs p38-mediated FOXO1 phosphorylation and glucagon-induced blood glucose increase. However, cAMP/PKA pathway is enhanced in C3GKO^Alb^ hepatocytes (Fig. 5A), promoting PKA-dependent actions in the liver such as phosphorylation of PYGL or expression of gluconeogenic enzymes (Supplementary Fig. 6C and Fig. 4B, C, respectively). The impaired insulin response of hepatocytes lacking C3G (evidenced by defective Akt and ERKs activation) could contribute to enhance cAMP/PKA dependent glucagon effects by preventing the decrease in cAMP levels, normally induced by insulin through phosphodiesterase phosphorylation by Akt [47]. Curiously, this defective insulin signaling detected in C3GKO^Alb^ hepatocytes is not due to impaired IR tyrosine phosphorylation or reduced expression (Fig. 5C). In fact, IR protein levels increased upon insulin stimulation, suggesting a defective c-Cbl-mediated IR degradation [48], supported by the fact that C3G participates in c-Cbl-dependent c-Mpl degradation in platelets [49]. An altered IR cellular trafficking like that observed for EGFR in C3G-silenced glioblastoma cells [24] could also explain it.

Notably, serum insulin and glucagon levels under feeding conditions tended to be increased in C3GKO^Alb^ mice compared to wt mice and associated with a decrease in blood glucose. Although these data do not fit in with the classical view of an inverse regulation of insulin and glucagon production and secretion, more recent data supports that glucagon and insulin are partners that collaborate to maintain metabolic homeostasis [10]. Hence, glucagon could facilitate glucose clearance even when high blood insulin levels are present. In that case, glucose could be used for anabolic pathways such as fatty acid synthesis in the liver of C3GKO^Alb^ mice, thanks to the enhanced expression of ACC and FAS. Additionally, the reduced blood glucose levels of C3GKO^Alb^ mice and the enhancement of glucagon-induced cAMP/PKA pathway in hepatocytes would favor ketogenesis and gluconeogenesis in ad libitum-fed or fasted C3GKO^Alb^ mice.

The upregulation of PKM2 in hepatocytes lacking C3G could also contribute to disrupting liver metabolism. High PKM2 levels accompanied by increased levels of LDHA would increase glycolysis, as evidenced by the higher lactate concentrations found in hepatocyte culture medium (Supplementary Fig. 5C) and the blood of C3GKO^Alb^ mice. This lactate being the primary source of carbon for tricarboxylic acid cycle in all tissues except the brain [50]. PKM2 upregulation could also enhance lipogenesis [51] and glucose clearance from blood through the activation of SREBP1c [51, 52] as reported in HCC cells. Therefore, although the response to insulin is very limited in the liver of C3GKO^Alb^ mice, this mechanism might facilitate a selective insulin sensitivity/resistance that allows SREBP1c activation [53].

Concerning the mechanism involved in C3G-dependent regulation of PKM2, we found that C3G absence or downregulation induces the upregulation of PTBP1 splicing factor, which promotes PKM2 expression (Fig. 3J, K and Supplementary Figs. 4G and 5F), as previously described [54].

Regarding fasting adaptation, it is known that fatty acids are elevated in the blood, increasing their uptake by the liver [55]. They can be stored as triglycerides or oxidized generating acetyl-CoA, which can be converted into ketone bodies. The latter would enhance ketogenesis in fasted C3GKO^Alb^ mice facilitated by the increased levels of *Hmgcs2*.

In summary, we have uncovered C3G as a new key player in the liver, required for full differentiation and metabolic activity of hepatocytes. C3G is essential for correct insulin and glucagon signaling. Therefore, C3G could represent a new relevant regulator of metabolic diseases and liver pathologies like steatosis with clinical implications.

## Supplementary information

Supplementary information

Uncropped western-blots

## Data Availability

All data on which the conclusions of this manuscript are based have been included in this manuscript either as main material (text and figures) or Supplementary Information (figures, tables, and text).
