# Correction: Deletion of C3G in hepatocytes impairs full liver maturation and alters glucose homeostasis

**DOI:** 10.1038/s41419-025-08181-z

**Published:** 2025-11-06

**Authors:** Nerea Palao, Jaime Mancebo, Cristina Baquero, Minerva Iniesta-González, Mateo Cueto-Remacha, María Rodrigo-Faus, Alvaro Gutierrez-Uzquiza, Paloma Bragado, Ángel M. Cuesta, Aránzazu Sánchez, Carmen Guerrero, Almudena Porras

**Affiliations:** 1 Departamento de Bioquímica y Biología Molecular, Facultad de Farmacia, Universidad Complutense de Madrid, Madrid, Spain; 2 Instituto de Investigación Sanitaria del Hospital Clínico San Carlos (IdISSC), Madrid, Spain; 3 Centro de Investigación Biomédica en Red de Enfermedades Hepáticas y Digestivas (CIBEREHD-ISCIII), Madrid, Spain; 4 Instituto de Biología Molecular y Celular del Cáncer (IMBCC), Universidad de Salamanca-CSIC, Salamanca, Spain; 5 Instituto de Investigación Biomédica de Salamanca (IBSAL), Salamanca, Spain; 6 Departamento de Medicina, Universidad de Salamanca, Salamanca, Spain

**Keywords:** Biochemistry, Pathogenesis

Correction to: *Cell Death and Disease* 10.1038/s41419-025-08031-y, published online 07 October 2025

In this article, the figures appeared truncated in the PDF version of the article.

Correct Figure 1
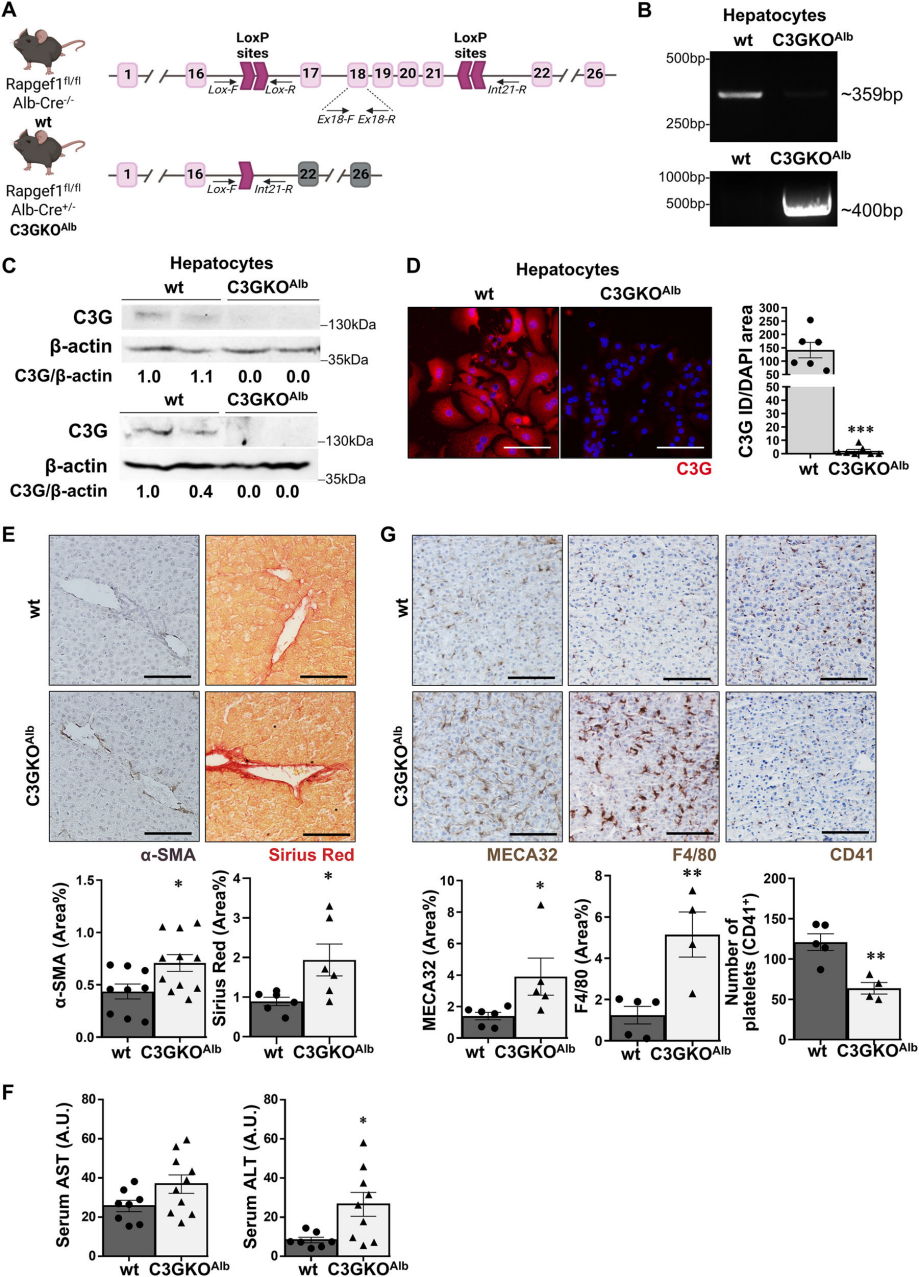


Correct Figure 2
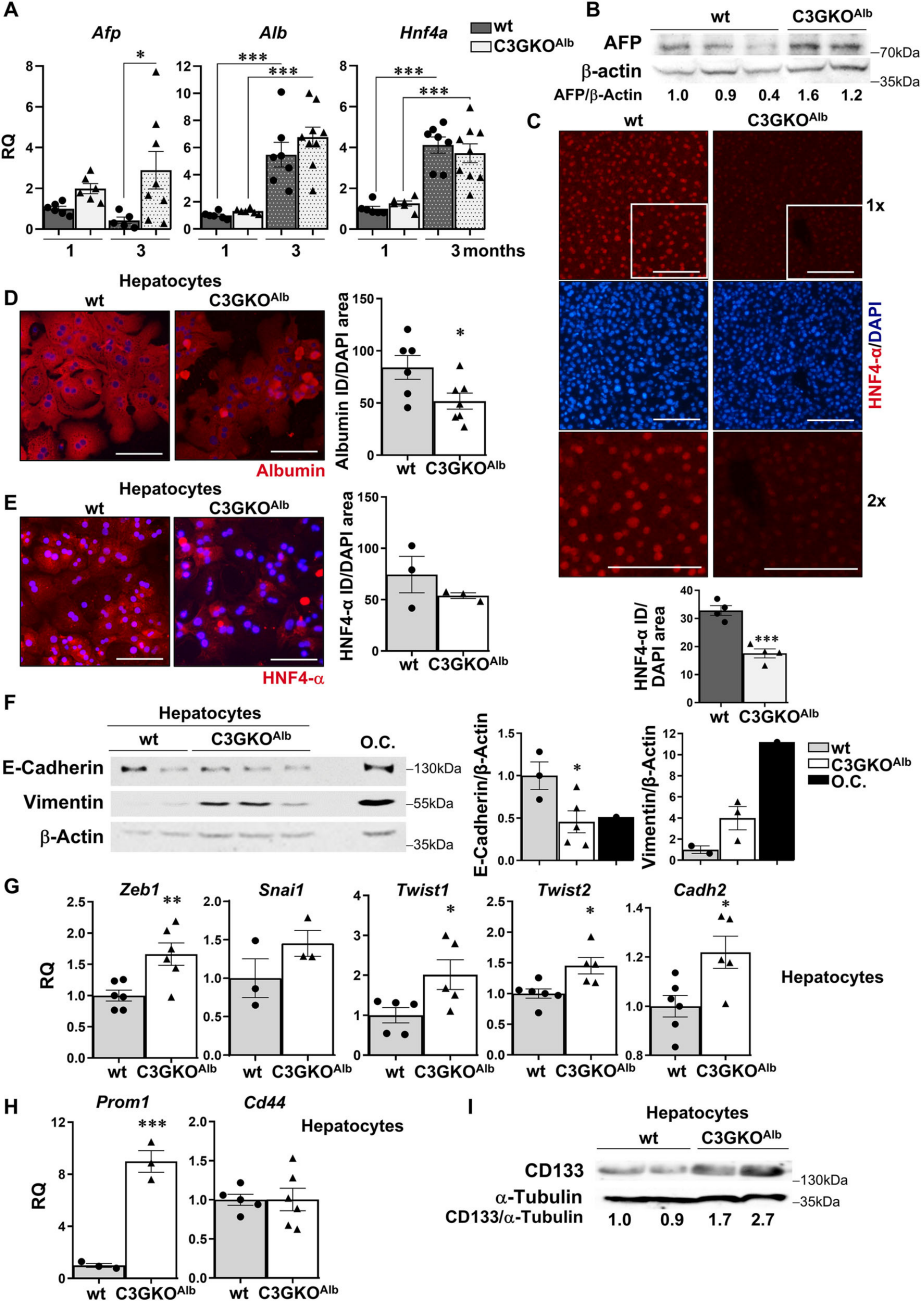


Correct Figure 3
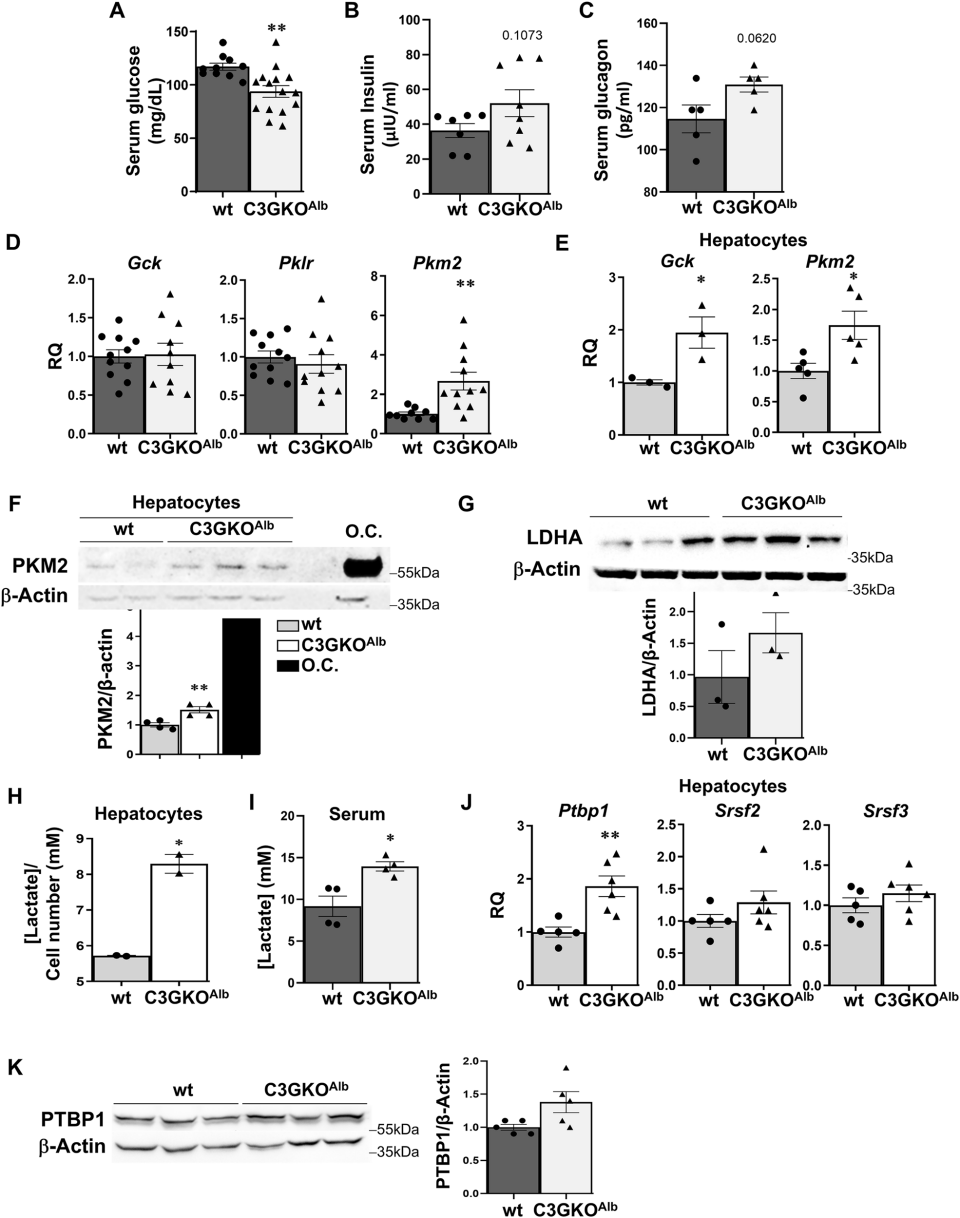


Correct Figure 5
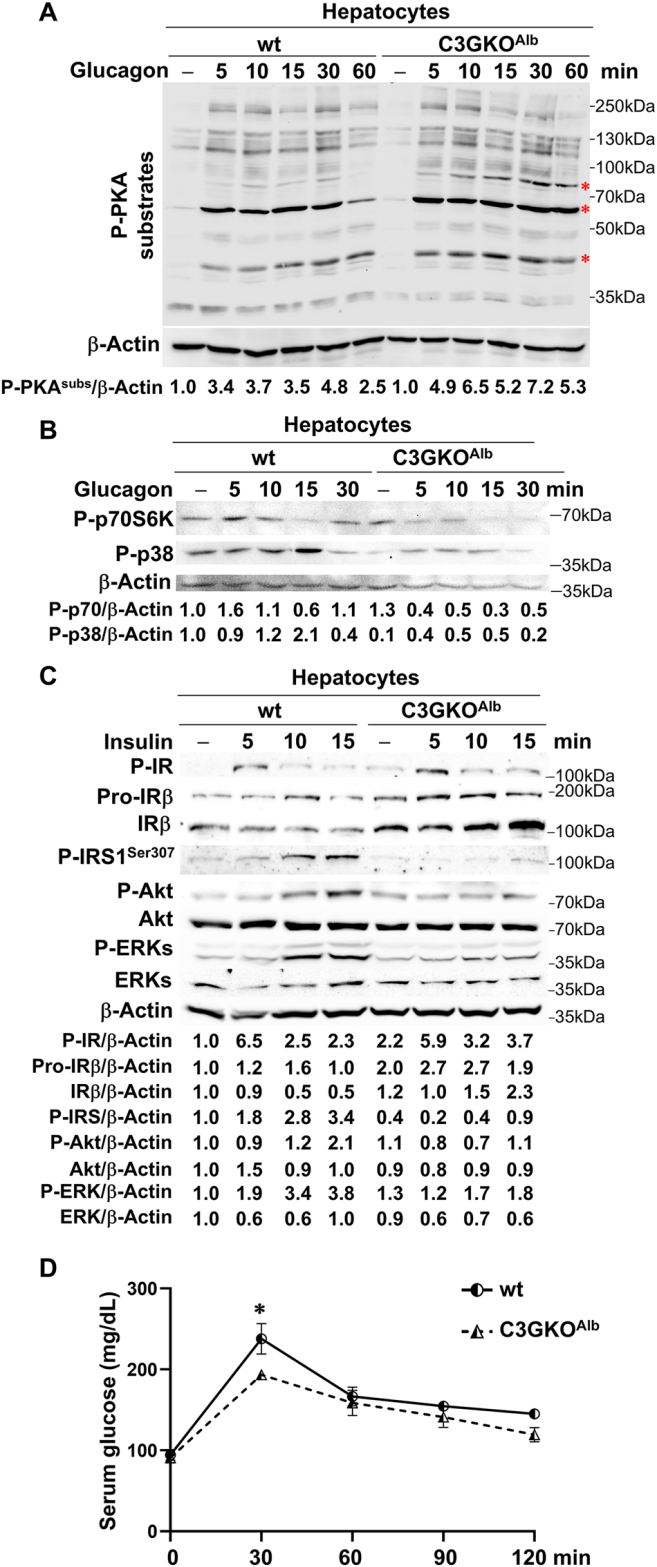


Correct Figure 7
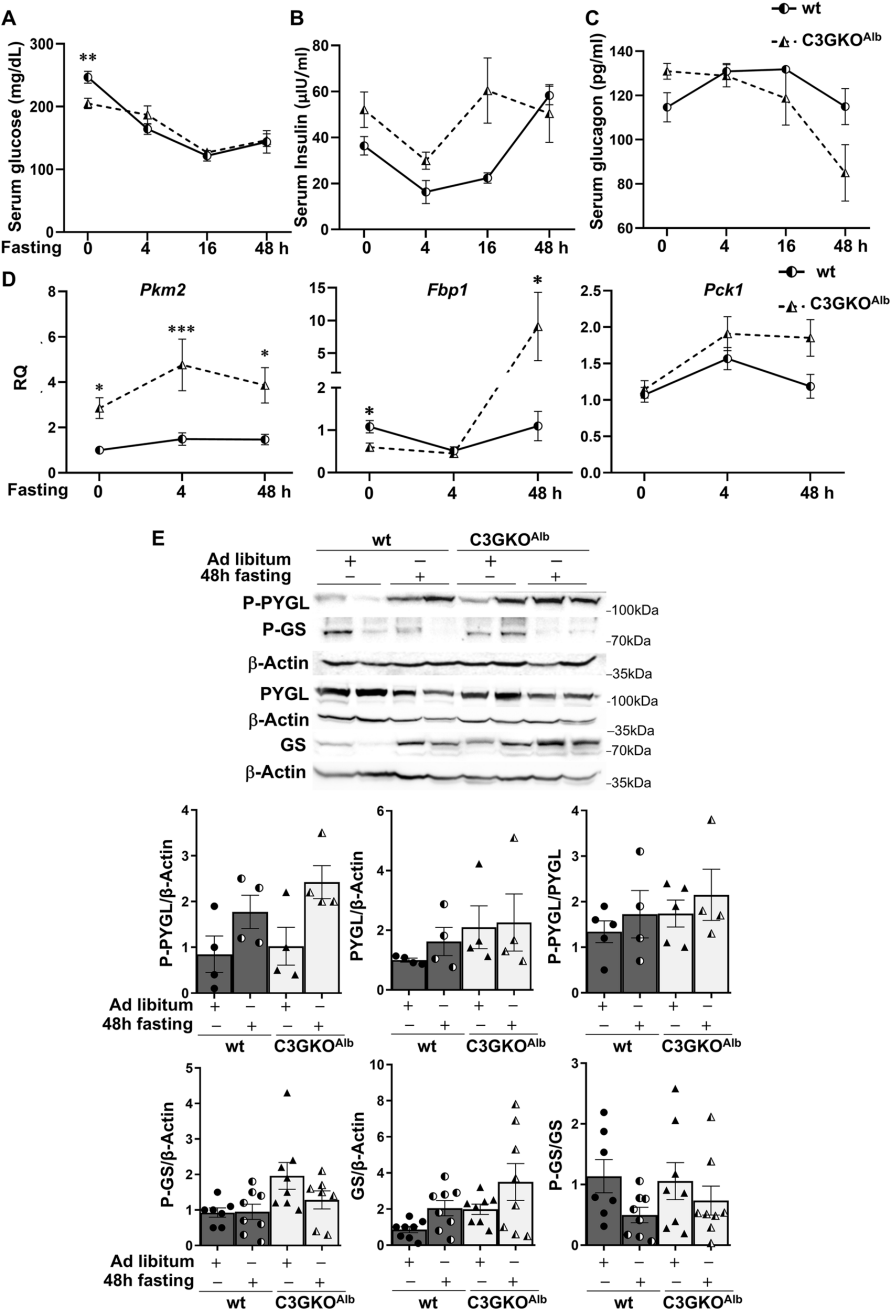


Correct Figure 8
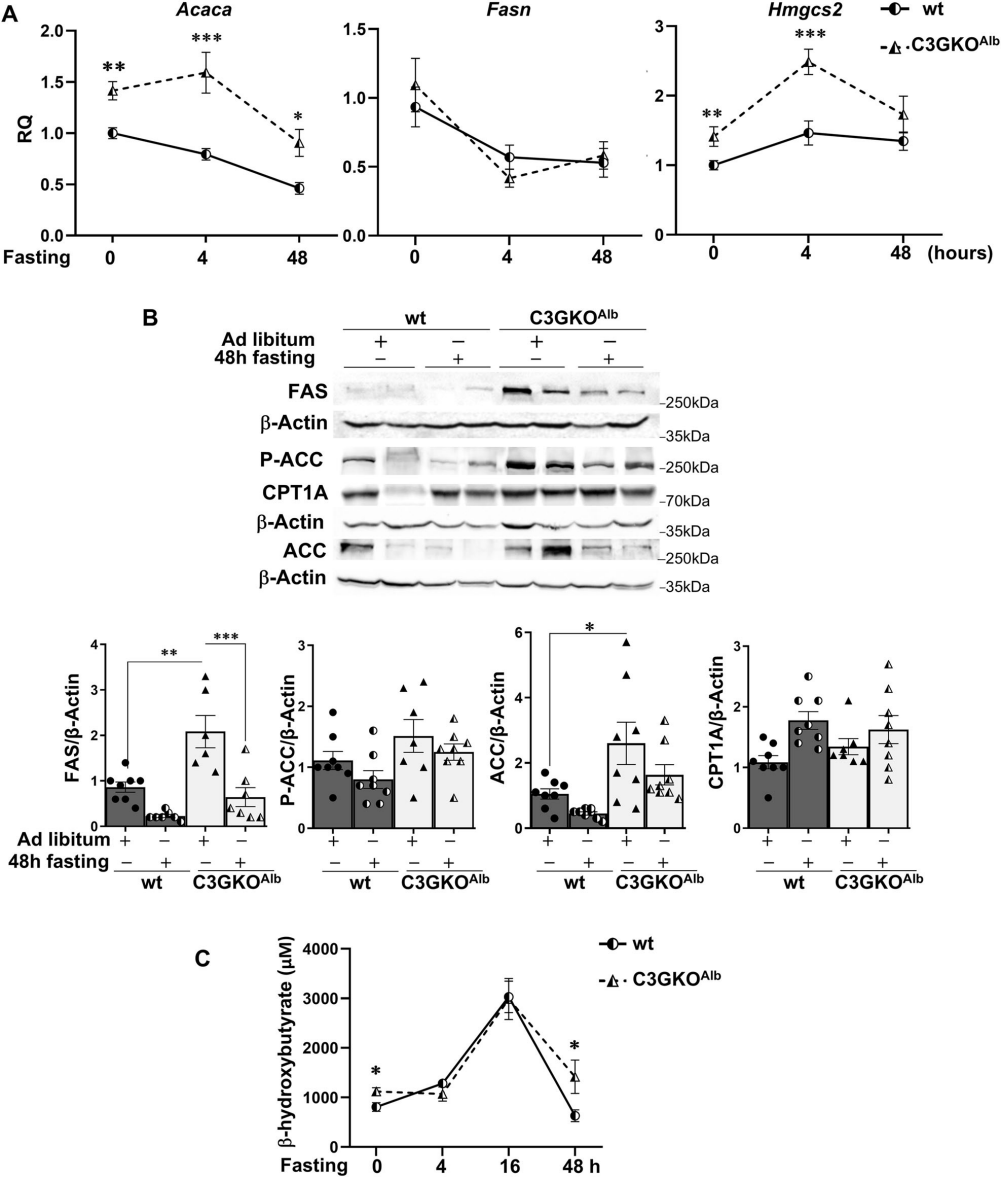


The original article has been corrected.

