# Supplementary material for: Deletion of C3G in hepatocytes impairs full liver maturation and alters glucose homeostasis
Source: Cell Death Dis. 2025 Oct 7;16(1):711. doi: 10.1038/s41419-025-08031-y (PMC12504625; doi:10.1038/s41419-025-08031-y)
Supplement: Supplementary file 1 — Supplementary information [file 41419_2025_8031_MOESM1_ESM.docx]

**Supplementary information**

It contains method details, 5 tables and 8 figures.

**Generation and genotyping of mice lacking C3G in hepatocytes**

Rapgef1^flox/flox^ mouse presents LoxP sites flanking exons 17–21, whose deletion was detected by PCR using genomic DNA (gDNA) from isolated hepatocytes and specific primers (Figure 1A and supplementary Table 1).

| **Supplementary Table 1. PCR primers to detect the deletion of the targeted exons** | |
| --- | --- |
| **Primer sequences (5’ 🡪 3’)** | |
| **Exon 18** | **C3G-Ex18-f** |
|  | CAGCACTCAGGTCCAACACA |
|  | **C3G-Ex18-r** |
|  | CAGAGCTCATCCACCACTCG |
| **Flanking LoxP sites** | **C3G-KO-LoxF** |
|  | AGCCTGTTGGCAAGTTTGG |
|  | **C3G-Int21R** |
|  | GGACTGGAGCATCTTTCAG |

Genotyping was performed using two different PCR reactions with tail DNA to detect floxed alleles and Cre recombinase, each amplified with specific primers (supplementary Table 2 and 3 and supplementary Figure 1) and DNA AmpliTools Master mix (Biotools, Madrid, Spain, 4557).

| **Supplementary Table 2. PCR protocol for the detection of the floxed alleles (genotyping)** | **Supplementary Table 3. PCR protocol for the detection of the Cre recombinase (genotyping)** |
| --- | --- |
| **Primer sequences (5’ 🡪 3’)** | **Primer sequences (5’ 🡪 3’)** |
| **C3G-KO-LoxF** | **Wild type Forward** |
| AGCCTGTTGGCAAGTTTGG | TGCAAACATCACATGCACAC |
| **C3G-KO-LoxR** | **Common Reverse** |
| CTGATGGAGAACCTAGCTGTGG | TTGGCCCCTTACCATAACTG |
|  | **Mutant Forward** |
|  | GAAGCAGAAGCTTAGGAAGATGG |

The PCR products were analyzed in 2% and 3% agarose gels, respectively, containing Gel Red Nucleic Acid Stain, and visualized in a VWR Imager Chemi Premium documentation system.

**Supplementary Figure 1. Illustrative PCR for Cre recombinase genotyping.** The PCR confirms the presence of Cre recombinase in Alb-C3GKO mice (390 bp) together with the presence of the wt allele (351 bp) in wt and heterozygous mice.

**Fasting induction** **and glucose tolerance test**

To perform metabolic studies, one- or three-month-old mice were fasted for different periods of time (4, 16 and 48 h). Mice (wt and C3GKO^Alb^) were randomly divided into two groups (10 mice per condition): subjected to food deprivation or fed *ad libitum* (all mice had free access to water). Fasting was performed overnight, during the hours of mice active feeding, except for the 4 h fasting, which was performed very early in the morning coincident with the last hours of feeding.

For glucose tolerance test, glucose (2g/Kg) was injected i.p. to 16h-fasted mice (wt and C3GKO^Alb^ (6 mice per condition) and blood glucose was measured by tail bleeding at 0, 30-, 60-, 90- and 120-min using glucose strips (ACCU-CHEK Aviva 06453970).

All animal experiments were carried out in compliance with the European Community Council Directive (2010/63/EU) and following guidelines for animal research from Complutense University Ethical Committee, approved by Comunidad de Madrid (Spain) (PROEX 226.25-21, PROEX 198.3/22 and PROEX 176.0/24).

The number of mice used in each experiment has been calculated using statistical methods (Anova and a p value=5) considering the variability of each experiment according to literature.

For all the experiments using mice the investigator was blinded to the group allocation during the experiment and when assessing the outcome.

**Analysis of plasma glucose, transaminases, insulin, glucagon, beta-hydroxybutyrate and lactate**

Plasma glucose levels were quantified using a Glucose-TR kit (Spinreact, 1001190). Glucose is oxidized by glucose oxidase, coupled to a peroxidase reaction in the presence of amino-phenazone. Absorbance at 505 nm was measured, and glucose levels were calculated using a glucose standard.

Plasma AST and ALT levels were quantified (in samples from at least 7 mice) using kits for measuring their activities (Spinreact, 41273 and 1001172, respectively), following the manufacturer’s instructions. In both cases, the rate of decrease in NADH concentration, measured photometrically at 340 nm for 3 minutes, is proportional to the catalytic concentration of AST and ALT present in the sample.

Plasma insulin levels were measured (in samples from at least 7 mice) using a mouse ELISA kit (Thermo Fisher Scientific, EMINS), according to the manufacturer’s instructions. In brief, samples and standards were incubated overnight at 4ºC in wells pre-coated with a mouse anti-insulin antibody, allowing the antigen binding. Next, wells were washed, and a biotin-conjugated anti-insulin antibody was added for 1 h at room temperature. After washing, streptavidin-horseradish peroxidase (SA-HRP) solution was added and incubated for 45 minutes at room temperature. Finally, wells were washed, incubated with TMB (3,3′,5,5′-Tetramethylbenzidine) substrate for 30 minutes in the dark and then, the reaction was stopped by adding the stop solution. Absorbance was measured at 450 nm within 30 minutes, and insulin levels were calculated using a standard curve.

Plasma glucagon levels were measured (in samples from at least 5 mice) using a mouse EIA kit (Sigma RAB0202), according to the manufacturer’s instructions. Briefly, an anti-Glucagon antibody was incubated overnight at 4ºC in wells pre-coated with an anti-rabbit secondary antibody. After washing, standards and samples to which 40 pg/mL of biotinylated glucagon were added and incubated for 2,5 hours at room temperature. The biotinylated glucagon peptide and the glucagon from the standard and the samples compete to interact with the glucagon antibody. After washing, SA-HRP solution was added and incubated for 45 minutes at room temperature. Finally, wells were washed, incubated with TMB substrate for 30 minutes in the dark and then, the reaction was stopped by adding the stop solution. Absorbance was measured at 450 nm immediately, and glucagon levels were calculated using a four-parameter logistic regression model.

Beta-Hydroxybutyrate was measured in serum of ad libitum-fed and fasted mice (at least 7 mice per genotype) at 4, 16 and 48 h using a colorimetric Kit (Cliniscience, Nanterre, France, MA-BHB-1) following the protocol provided by the company.

Lactate was determined in hepatocyte condition media (from 2 hepatocyte cultures coming from two different mice) and mice serum from ad libitum-fed mice (6 per genotype) using lactate dehydrogenase (Roche, 10127876001, Basel, Switzerland) for the conversion of lactate to pyruvate measuring NADH generation from NAD^+^ spectrophotometrically at 340 nm.

**Immunohistochemistry, collagen and glycogen analysis in liver sections**

Liver samples (coming from at least 5 mice per genotype) were fixed in 4% PFA o/n were embedded in paraffin. Sections (7 µm) were mounted into APES ((3-Aminopropyl) triethoxysilane) pre-coated slides. After deparaffinization and rehydration, antigen retrieval with citrate buffer (10 mM, pH=6) was performed. Then, sections were treated as follows for immunohistochemistry (IHC): ethanol 90%; ethanol 100%; 3% H_2_O_2_ in methanol 100%; ethanol 100%; ethanol 90% to block endogenous peroxidase and washed with H_2_O and PBS. Next, slides were permeabilized with 0,5% Triton X-100-PBS, washed with PBS and incubated in blocking buffer (3% BSA-1,5% normal goat serum-PBS (Invitrogen, Carlsbad, CA, USA, PCN5000)) 1h at RT, washed with PBS and incubated with primary antibodies against: α-SMA (Dako, Glostrup, Denmark, M0851; 1:50), F4/80 (Biorad, Hercules, CA, USA, MCA497; 1:2), CD41 (Abcam, Cambridge, UK, ab181582; 1:50), and MECA32 (CST, Danvers, MA, USA, 24764;1:25) in blocking buffer overnight at 4ºC in a wet chamber. After washing with PBS, sections were incubated with the corresponding biotinylated secondary antibody (anti-rabbit (Vector, Newark, CA, USA, BA-1100), anti-mouse (Vector BA-2000) or anti-rat (Vector BA-4000)) for 1h at RT. Then, they were washed with PBS, incubated with avidin/biotin (1:1; Vector PK-6100) for 30 min at RT in dark, washed with PBS and revealed with DAB (Vector SK-41000). Then, they were washed with H_2_O, incubated with hematoxylin (2 min) and washed with H_2_O. Sections were then dehydrated and mounted using DPX. Images (20/sample) were taken using an Eclipse TE300, Nikon microscope coupled with a Nikon Digital Sight DS-U2 camera (20X) or a Leica DMC 4500 microscope. Then, α-SMA, F4/80 and MECA32 were quantified by measuring the DAB-positive area using ImageJ software (Color deconvolution – H&E DAB). CD41-positive cells were manually counted in representative fields at 20× magnification.

Sirius Red staining was used to detect liver collagen. Dewaxed and rehydrated liver paraffin sections were incubated with 0,1% Picro-Sirius Red (Direct Red 80, Sigma 365548) in picric acid (Sigma P6744-1GA). Then, tissues were washed twice with acidified water (0.5% Glacial acetic acid in water) and mounted using DPX. Sirius Red positive area was determined using the threshold tool to measure the stained collagen fibers.

Periodic Acid (PA) Schiff staining was used to detect liver glycogen in sections. Tissues were deparaffinized and re-hydrated. Then, tissues were incubated in a 0.5% PA solution, washed with water, incubated with Schiff’s Reagent (Panreac, Barcelona, Spain, 171588) and washed with water. As a nuclear counterstaining, slides were incubated with Hematoxylin for 1 min. Finally, tissues were mounted using DPX. For collagen and glycogen quantification, 20 representative images per mouse were randomly taken using a phase-contrast microscope (Nikon Eclipse TE300). The positive area was quantified using ImageJ software. Glycogen content was quantified by measuring the PAS-positive area using ImageJ software (Color deconvolution – H PAS).

**Isolation, culture and stimulation of adult hepatocytes**

Hepatocytes were isolated (from 3 mice per genotype and experiment) using the classic two-step collagenase perfusion technique followed by isodensity purification in a Percoll gradient, as previously described (1,2). The liver of anesthetized mice was first perfused with warm Hanks Balanced Salt Solution containing 1 M Hepes and 0.2 mM EGTA, followed by perfusion with digestion solution containing Williams E (-) L-Glutamine culture medium, 1 M Hepes and Collagenase IV (Worthington, Lakewood, NJ, USA, LS004186). After the perfusion, the liver was placed into a dish with attachment media (DMEM: F12 high glucose w/o glutamine supplemented with 0.02% BSA, insulin-transferrin-selenium (ITS) (Gibco, Grand Island, NY, USA, 41400045) and 5 mM sodium pyruvate. The resulting cell suspension was filtered through a 70 µm cell strainer (Corning, Corning, NY, USA, CLS431751). Cells were centrifuged (500 rpm, 5 minutes, 4 ºC) and the supernatant was discarded. The pellet was resuspended in attachment media and viable hepatocytes were isolated using a Percoll (Cytiva, Marlborough, MA, USA, 17-0891) gradient. After centrifugation (1,000 rpm, 10 min, 4ºC), the pellet contained alive purified hepatocytes. Cell suspension was centrifuged again (500 rpm, 5 min, 4 ºC) and the pellet was resuspended in attachment media. Cells were seeded on collagen I (Roche, Basel, Switzerland, 11179179001) pre-coated plates. All the experiments were performed within 24 h after plating to avoid hepatocyte dedifferentiation. Prior to treatments with 10 nM insulin (Gibco, 12585014) and 20 nM glucagon (Sigma, G2044), hepatocytes were maintained in attachment media deprived from FBS and ITS for 3h.

**Immunofluorescence analysis in adult hepatocytes**

Hepatocytes (from at least 3 mice per genotype) were fixed with cold methanol (-20 ºC) for 2 min on ice, or 4% PFA (Sigma Aldrich, 158127) (20 min), and permeabilized with 0.5-1% Triton X-100 in PBS and 0.1% SDS in PBS (in the case of nuclear proteins (like HNF4α)). Next, coverslips were washed twice with PBS and blocked with 3% BSA -1.5% NGS (normal goat serum) (or 4.5% NGS for Albumin staining) in PBS for 1 h at room temperature. In both cases, cells were incubated overnight at 4 ºC with primary antibodies against: Albumin (Nordic, Täby, Sweden, RARA-ALB; 1:50), C3G (N-terminal, Genosphere, Paris, France custom made; 1:100), CC3 (CST, 9661S), CD133 (Miltenyi, Bergisch Gladbach, Germany, 130-092-442, 1:100), HNF4-α (Abcam, ab181604; 1:50), E-cadherin (BD, Franklin Lakes, NJ, USA, 610182; 1:50) and PKM2 (CST, 3198S; 1:25) diluted in blocking solution. Coverslips were washed with PBS and incubated with secondary antibodies: goat anti-rabbit, 555 nm (Invitrogen, A32732) or goat anti-mouse 555 nm (Invitrogen, A32727), 1:200 (1h) and DAPI (4′,6-diamidino-2-phenylindole) (Panreac, A4099; 1:1000) diluted in blocking buffer. After washing with PBS, cells were mounted using Prolong Gold Antifade Reagent (Invitrogen, P36930).

Cells were visualized in either a Nikon Eclipse TE300 epifluorescence microscope or a Leica DMC 4500 microscope. Images were taken at the same exposure time. Fluorescence quantification was performed using ImageJ software, using the threshold tool to identify positive areas. Fluorescence intensity was measured in at least 8 fields per condition and represented by the Integrated Density (ID) parameter (ID = Mean value of fluorescence intensity x positive area percentage) referred to DAPI area or cell number.

**RNA isolation, RT-qPCR analysis and RT-PCR evaluation of PKM1 and PKM2**

Total RNA isolation and RT-qPCR analysis was performed as previously described (3). Briefly, total RNA was isolated using NucleoSpin RNA kit (Macherey-Nagel, Düren, Germany, #740955.50) and reverse transcribed using SuperScript IV-RT kit (Invitrogen) or SuperScript III-RT kit (Invitrogen). cDNA was amplified using the following specific primers for *Acca,* *Afp, Alb, Cdh2, Cd44, Fasn, Fbp1, G6Pc, Gcgr, Gck, Gys1, Hmgcs2, Hnf4a, Insr, Pck1, Pklr, Pkm2, Prom1, Ptbp1, Pygl, Snai1, Srsf2/3, Twist1/2, Zeb1* and *Gusb* to normalize (supplementary Table 4). ΔCt (Ct (threshold cycle) for a specific gene minus Ct for *Gusb*) was calculated and then, referred to non-silenced control values (sample ΔCt-non-silenced ΔCt= ΔΔCt) to calculate RQ (2^-ΔΔCt^).

| **Supplementary Table 4. Sequences of primers used for RT-qPCR** | | | |
| --- | --- | --- | --- |
| **Protein** | **Mouse**  **gene** | **Forward primer**  **(5‘🡪3’)** | **Reverse primer**  **(5‘🡪3’)** |
| ACCα | *Acaca* | CTGCAGAAACTCATCCTCTCG | TCGAACATACACCTCCAGAGC |
| AFP | *Afp* | TGTTGCCAAGGAAACTCG | GCAGCACTCTGCTATTTTGC |
| Albumin | *Alb* | ATCTGCACACTTCCAGAGAAG | TCCATGACAGTCTTCAGTTGC |
| CD133 | *Prom1* | CTGGGATTGTTGGCCCTCTC | AGGGCAATCTCCTTGGAATCA |
| CD44 | *Cd44* | GGCCACCATTGCCTCAACTGT | TGCACTCGTTGTGGGCTCCTG |
| FAS | *Fasn* | GGATGGCCGCGGTTTAAATA | CCTCCATGGCTCTTCTCTGTC |
| FBPase | *Fbp1* | GCATCGCACAGCTCTATGGT | ACACAGGTAGCGTAGGACGA |
| G6Pase | *G6pc* | CTGGAGTCTTGTCAGGCATT | AATCCAAGCGCGAAACCAAA |
| GCGR | *Gcgr* | AGATCGAGGTCCAGAAGGGG | AGACTGTAGCCCACGGTGTA |
| GCK | *Gck* | GGGAACAACATCGTGGGACT | CCTCACATTGGCGGTCTTCA |
| GUSB | *Gusb* | AAAATGGAGTGCGTGTTGGGTCG | CCACAGTCCGTCCAGCGCCTT |
| GYS1 | *Gys1* | ACATCACCACCAACGACGGA | TAGCCGATCCCTCTCAGCCT |
| HMGCS2 | *Hmgcs2* | AGTGGAAGCAAGCTGGAAAC | ATCAACCGAGCCAGGGATTT |
| HNF4α | *Hnf4α* | GGCATGGATATGGCCGACTAC | TTCAGATGGGGACGTGTCATT |
| INSR | *Insr* | AGGAGCTGGAGGAGTCTTCAT | TGCCTGAAGAGGTTTTTCTGGG |
| N-cadherin | *Cdh2* | ACTGGGTCATCCCGCCAATCA | TGAAGATGCCCGTTGGAGGCTG |
| PEPCK | *Pck1* | TGGAAGGTCGAATGTGTGGG | AGCCCTTAAGTTGCCTTGGG |
| PKLR | *Pklr* | CGGGCTCATCTCCTTAGTGG | GGCAAGTTCACACCCTTCCT |
| PKM2 | *Pkm2* | TATCGCAGCAGGAACCGAAG | GCATGGTTCCTGAAGTCCTTTG |
| PTBP1 | *Ptbp1* | GTCGGTACAAAGCGGGGAT | CGTTTCCATTGGCTGCTGAG |
| PYGL | *Pygl* | CCAAGTTCGGCTCCAAGGAT | AGCTGGATGGCTACCTGATCT |
| Snail1 | *Snai1* | TCCAAACCCACTCGGATGTGAAGA | TTGGTGCTTGTGGAGCAAGGACAT |
| SRSF2 | *Srsf2* | CTCGCTTAGTTGCTGCTCAG | CTCGGTAGCTACAGTCCTCG |
| SRSF3 | *Srsf3* | CGGAGCGTTAGGATTTGAGC | TCCAAGGGACAGGAATCACG |
| Twist1 | *Twist1* | CCGGAGACCTAGATGTCATTGT | CCACGCCCTGATTCTTGTGA |
| Twist2 | *Twist2* | GCAAGAAGTCGAGCGAAGAT | GCTCTGCAGCTCCTCGAA |
| Zeb1 | *Zeb1* | GTACAAACACCACCTGAAAGAGC | CCATTCACAGGCATCAAGC |

A semiquantitative analysis of *PKM1* and *PKM2* in HLE HCC cells was performed by RT-PCR. cDNA was amplified using specific primers for human *PKM1* (f-GAGCATGATCAAGAAGCCCC and r-GGCTCGCACAAGTTCTTCAA) and *PKM2* (f-GAGCATGATCAAGAAGCCCC and r-AAGTGGTAGATGGCAGCCTC) normalizing with *GUSB* (f-ATCACCGTCACCACCAGCGT and r-GTCCCATTCGCCACGACTTT).

**Western-blot analysis**

Western-blot analysis was carried out as previously described (3). Membranes were probed with primary antibodies against: ACC, P-ACC, AFP, Akt, P-Akt C3G, CD133, CPT1A, E-cadherin, ERK, P-ERKs, FAS, GS, IRβ, P-IRβ, P-IRS, P-GS, P-p38, P-p70S6K, P-PKA substrates, PKM2, PTBP1, PYGL, P-PYGL, Vimentin and β-Actin or α-Tubulin to normalize (supplementary Table 5).

| **Supplementary Table 5. Primary antibodies used for western blot** | | | | |
| --- | --- | --- | --- | --- |
| **Antibody** | **Laboratory and reference** | **Isotype** | **Dilution** | **Molecular weight (kDa)** |
| ACC | CST, 3676S | Rabbit | Rabbit | 280 |
| P-ACC | CST, 3661 | Rabbit | 1:1000 | 280 |
| ACC | CST, 3676S | Rabbit | 1:1000 | 280 |
| β-Actin | CST, 3700 | Mouse | 1:1000 | 45 |
| AFP | SCBT, Dallas, TX, USA, 130302 | Mouse | 1:500 | 68 |
| P-Akt (Ser473) | CST, 9271 | Rabbit | 1:1000 | 60 |
| Akt | CST, 9272 | Rabbit | 1:1000 | 60 |
| C3G C19 | SCBT, 869 | Rabbit | 1:1000 | 130 |
| C3G F5 | SCBT, 376992 | Mouse | 1:1000 | 130 |
| CD133 | Miltenyi, 130-092-442 | Rat | 1:250 | 120 |
| CPT1A | Proteintech, Rosemont, IL, USA,15184-1-AP | Rabbit | 1:1000 | 88 |
| E-Cadherin | BD, 610182 | Mouse | 1:1000 | 130 |
| P-ERKs (Thr202/Tyr204) | CST, 9101 | Rabbit | 1:1000 | 42/44 |
| ERKs | CST, 9102 | Rabbit | 1:1000 | 42/44 |
| FAS | CST, 3189 | Rabbit | 1:1000 | 273 |
| P-GS | CST, 3891S | Rabbit | 1:1000 | 84 |
| GS | CST, 3886S | Rabbit | 1:1000 | 84 |
| P-IR^Tyr1345^ | CST, 3026 | Rabbit | 1:1000 | 95 |
| IRβ | CST, 3025 | Rabbit | 1:1000 | 95 |
| P-IRS1^Ser307^ | Upstate cell signaling solutions, Burlington, MA, USA, 07-247 | Rabbit | 1:1000 | 132 |
| P-p38MAPK (Thr180/  Tyr182) | CST, 9211 | Rabbit | 1:500 | 38 |
| p38MAPK (C-20) | SCBT, 535 | Rabbit | 1:1000 | 38 |
| P(Thr389)-p70S6K | CST, 9205S | Rabbit | 1:1000 | 70 |
| P-PKA substrates | CST, 9621 | Rabbit | 1:1000 | several |
| PKM2 | CST, 3198S | Rabbit | 1:500 | 60 |
| PTBP1 | CST, 57246S | Rabbit | 1:2500 | 57 |
| P-PYGL | Thermo Fisher, PA5-114628 | Rabbit | 1:1000 | 97 |
| PYGL | Thermo Fisher, PA5-76996 | Rabbit | 1:2500 | 97 |
| α-Tubulin | CST, 2146 | Mouse | 1:1000 | 55 |
| Vimentin | BD, 550513 | Mouse | 1:500 | 57 |

**Transient PTBP1 silencing in hepatocellular carcinoma cells**

Transient PTBP1 silencing was performed in the human HLE hepatocellular carcinoma (HCC) cell line (Runtogen, Louisville, CO, USA, RWT-744) with or without permanent C3G silencing (3 independent replicas). First, 125 pmol PTBP1 RNAi (Dharmacon, Lafayette, CO, USA, L-003528-00-0005) were diluted in 500 μl Opti-MEM® I Medium without serum (Fisher scientific, 31985070) using in parallel 125 pmol control RNAi (Dharmacon #D-001810-10-05) as a control. Next, Lipofectamine™ RNAiMAX (7.5 μl) (Fisher scientific, 10514953) was added, mixed and incubated for 30 minutes at room temperature. Then, cells diluted in complete growth medium without antibiotics (250,000 cells/well) were added to RNAi-Lipofectamine™ RNAiMAX complexes and mixed. After 96 hours of incubation at 37°C in a CO_2_ incubator gene knockdown and PKM1/2 expression were analyzed.

Mycoplasma was tested routinely by PCR using cell medium.

**Statistical analysis**

Data were represented as the mean values ± S.E.M (standard error of the mean) of, at least, 3 independent experiments. Unpaired Student’s t-test was used to compare two experimental groups under normal data distribution. One-way or two-way ANOVA analyses were used to compare more than two groups with one or two variables, respectively, followed by a multiple comparison Bonferroni test. All statistical analyses were carried out with GraphPad Prism Software version 8.0.1. Statistical significance was considered when p value ≤0.05.

Outliers were identified and removed only when statistically justified using GraphPad's Outlier Calculator, which applies Grubbs’ test, to detect whether the most extreme value in a dataset significantly deviates from the rest.

**Supplementary Figure 2-Effect of C3G deletion in hepatocytes on Epac1, Epac2 and Rap1 expression and liver morphology.** A) Western-blot analysis of Epac1 and Epac2 protein levels in livers from C3GKO^Alb^ and wt mice normalized with β-actin. Histograms show the quantification of Epac1 or Epac2 normalized with β-Actin and referred to wt values. B) Western-blot analysis of Rap1 protein levels in livers from C3GKO^Alb^ and wt mice normalized with β-actin and referred to wt values. C) Liver morphology of C3GKO^Alb^ and wt mice. Top panels, images of livers. Lower panels, hematoxylin/eosin staining (H&E) of paraffin liver sections from one-month old C3GKO^Alb^ and wt mice. D) Immunofluorescence analysis of cleaved-caspase 3 (CC3) in C3GKO^Alb^ and wt hepatocytes. **p≤0.01 compared to wt hepatocytes. Scale bars: 25 μm. n=2-6.

**Supplementary Figure 3-Hepatocytes lacking C3G show a less differentiated phenotype.** A) Immunofluorescence analysis of E-cadherin in hepatocytes isolated from C3GKO^Alb^ and wt mice. Nuclei were stained with DAPI. Histogram shows the quantification of E-cadherin versus DAPI area (%). B) Immunofluorescence analysis of CD133 in hepatocytes isolated from C3GKO^Alb^ and wt mice. Nuclei were stained with DAPI. Scale bars: 25 μm. **p≤0.01 compared to wt hepatocytes (n=3-7).


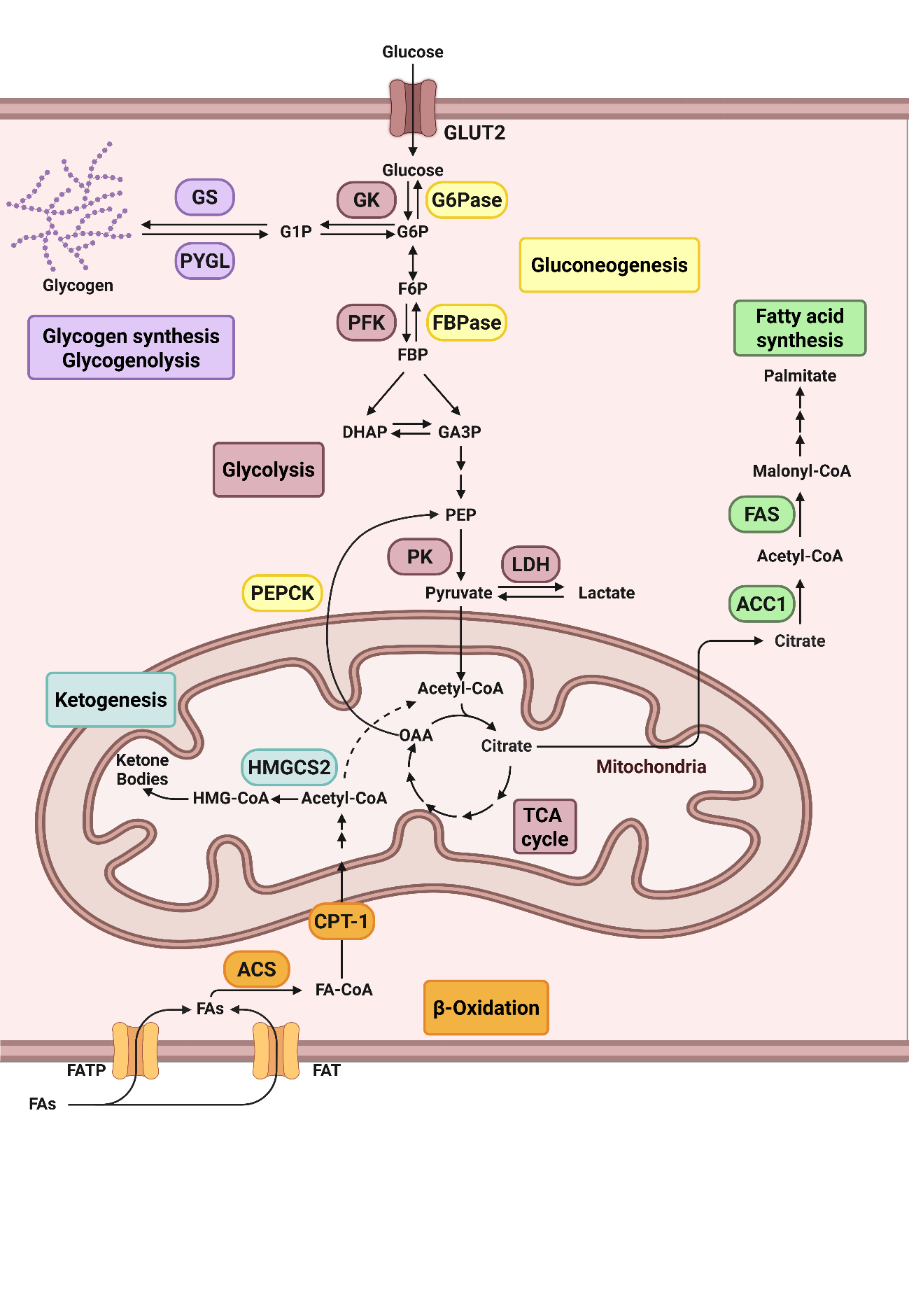


**Supplementary Figure 4-Scheme of the main liver metabolic pathways.** The scheme shows the main pathways involved in glucose and fatty acid (FA) metabolism in the hepatocytes including glycolysis, gluconeogenesis, glycogenolysis, glycogen synthesis, tricarboxylic acid cycle (TCA), FA synthesis and oxidation, as well as ketogenesis. The most important enzymes from each pathway are included: GK (glucokinase); PFK (phosphofructokinase); PK (pyruvate kinase); LDH (Lactate dehydrogenase); G6Pase (Glucose 6 phosphatase); FBPase (fructosebiphosphatase); GS (Glycogen synthase); PYGL (Glycogen phosphorylase); ACC1 (acetylCoA carboxylase); FAS (fatty acid synthase); ACS (Acyl Coa synthase); CPT-1 (Carnitine-palmitoyl transferase); HMGCS2 (Hydroxy-methyl-glutaryl CoA synthase). Created with BioRender.com.

**Supplementary Figure 5-C3G deletion in hepatocytes increases the expression of PKM2 and LDA glycolytic enzymes.** A) Immunofluorescence analysis of PKM2 in hepatocytes isolated from C3GKO^Alb^ and wt mice. Nuclei were stained with DAPI. Scale bars: 25 μm. Histogram shows the quantification of PKM2 versus DAPI area (%). B) RT-qPCR analysis of *Ptbp1, Srsf2* and *Srsf3* mRNA expression in livers from C3GKO^Alb^ and wt mice. *p≤0,05, **p≤0,01 compared to wt mice (n=2-12). C) Western-blot analysis of C3G and PKM2 protein levels in C3G silenced (shC3G) and parental HLE cells normalized with α-Tubulin and referred to values of non-silenced cells. D) Effect of PTBP1 silencing on C3G silenced (shC3G) and parental HLE cells. Western-blot analysis of PTBP1 and PKM2 protein levels normalized with α-Tubulin and referred to levels of non-silenced cells. E) RT-PCR analysis of *PKM1* and *PKM2* mRNA expression normalized with *GUSB* upon PTBP1 silencing in C3G silenced (shC3G) and parental HLE cells. Images of the electrophoretic analysis of the amplified fragments are shown. *p≤0.05, **p≤0.01 compared to wt mice (n=3-12).

**Supplementary figure 6-Effect of C3G deletion in hepatocytes on liver glycogen levels and metabolism.** A) Periodic Acid Schiff (PAS) staining of glycogen in liver sections. Histogram shows the quantification of the stained area (%). B) RT-qPCR analysis of *Pygl* and *Gs* mRNA expression in the liver of one-month-old C3GKO^Alb^ and wt mice. C) Left panel, western-blot analysis of PYGL, P-PYGL, GS and P-GS protein levels in livers from C3GKO^Alb^ and wt mice normalized with β-Actin. Right panel, histograms show the quantification of different western blots. *p≤0.05, **p≤0.01 compared to wt mice (n=2-10).

**Supplementary Figure 7-Effect of C3G deletion in hepatocytes on the liver expression of insulin and glucagon receptors.** RT-qPCR analysis of *Insr* and *Gcgr* mRNA expression in livers from C3GKO^Alb^ and wt mice (n=5). RQ (relative quantification).

**Supplementary Figure 8- Effect of C3G deletion on the expression of fatty acid metabolism enzymes in hepatocytes.** Hepatocytes isolated from C3GKO^Alb^ and wt mice were used. A) RT-qPCR analysis of *Acaca* and *Fasn* mRNA expression. B) Western-blot analysis of P-ACC and ACC protein levels normalized with β-Actin and referred to wt levels. Histograms show the quantification of P-ACC and ACC versus β-Actin. C) Western-blot analysis of FAS and CPT1A protein levels normalized with β-Actin or α-Tubulin, respectively. Histograms show the quantification of FAS versus β-Actin and CPT1A versus α-Tubulin. *p≤0.05 compared to wt mice (n=3-5).
