# Supplementary material for: Deletion of C3G in hepatocytes impairs full liver maturation and alters glucose homeostasis
Source: Cell Death Dis. 2025 Oct 7;16(1):711. doi: 10.1038/s41419-025-08031-y (PMC12504625; doi:10.1038/s41419-025-08031-y)

Figure 1C

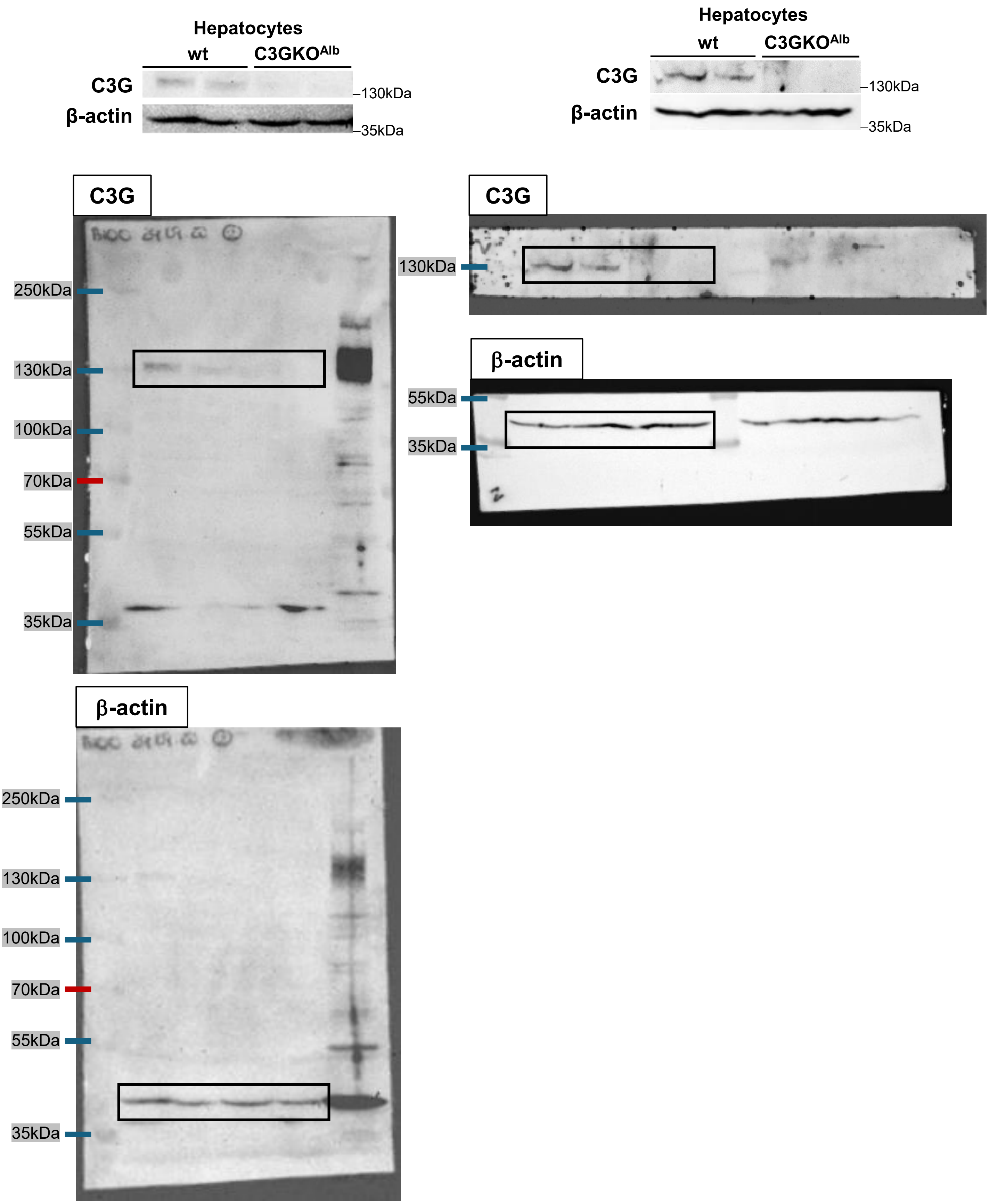
Hepatocytes
wt
C3GKOAlb
C3G
β-actin
130kDa
35kDa
Hepatocytes
wt
C3GKOAlb
C3G
β-actin
130kDa
35kDa
C3G
250kDa
130kDa
100kDa
70kDa
55kDa
35kDa
C3G
130kDa
β-actin
55kDa
35kDa
β-actin
250kDa
130kDa
100kDa
70kDa
55kDa
35kDa

Figure 2B

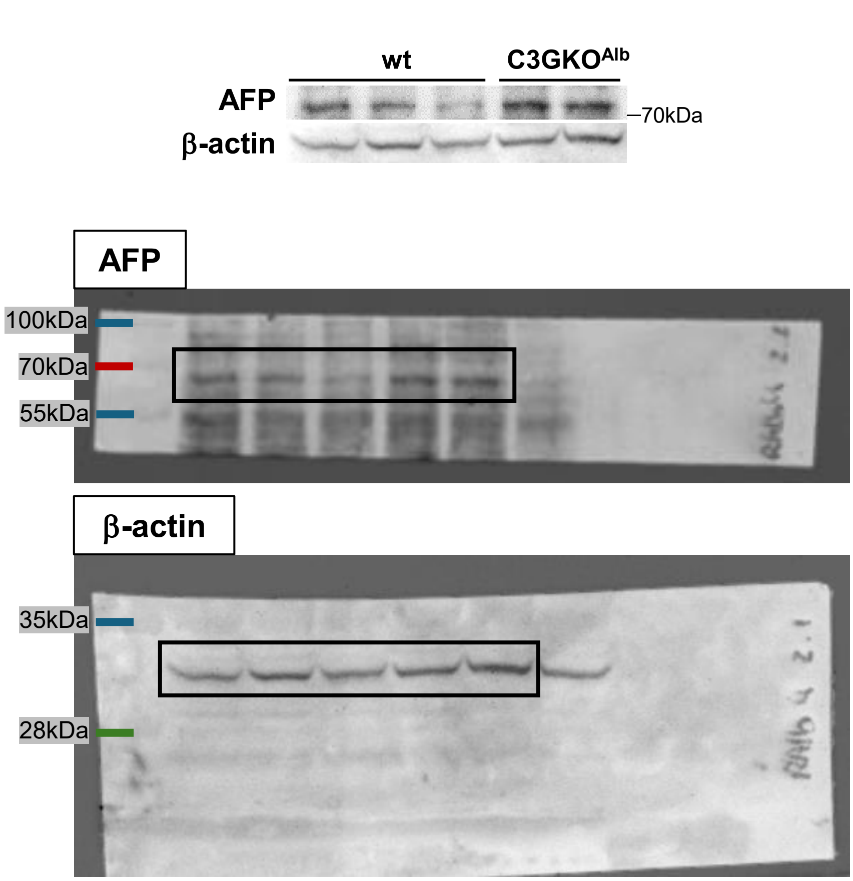

Figure 2F

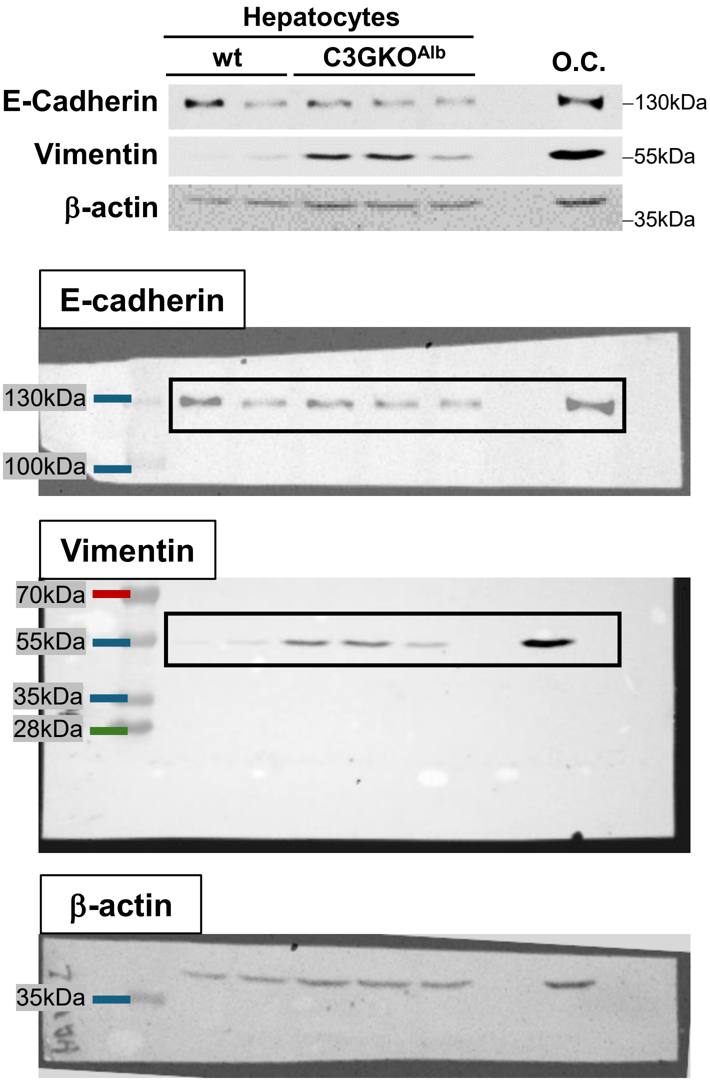

Figure 2I

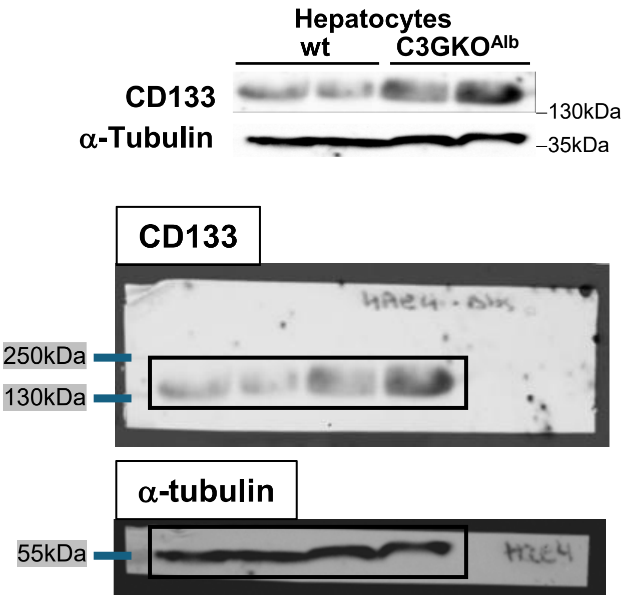

Figure 3F

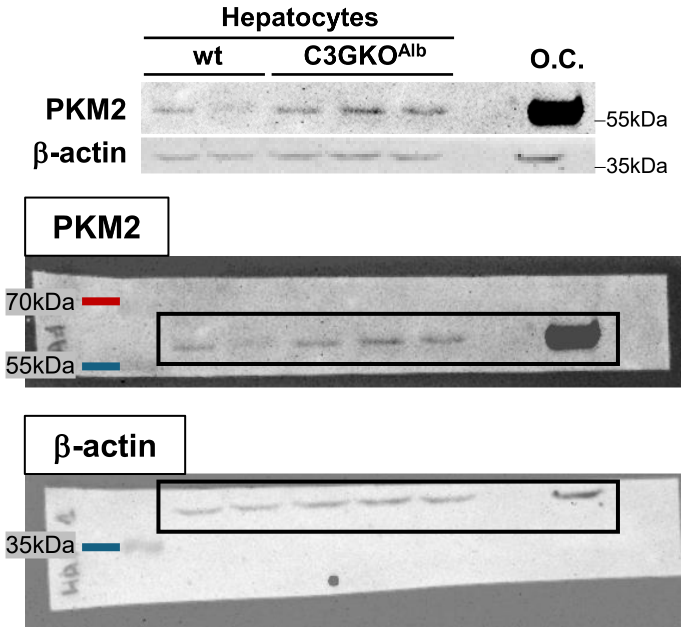

Figure 3H

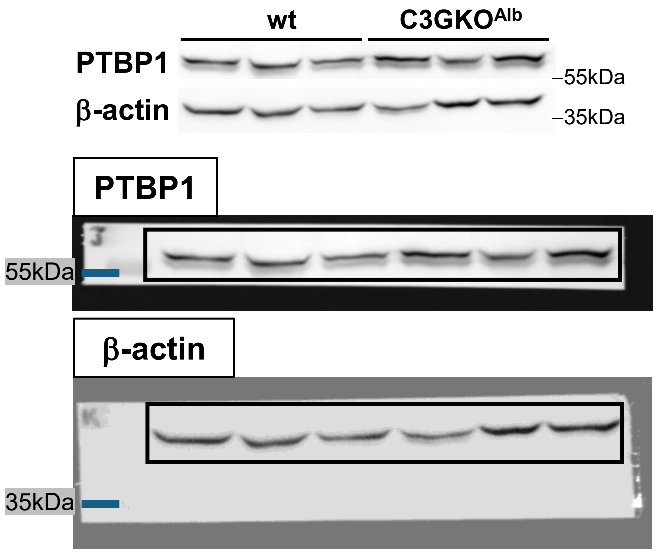

Figure 3I

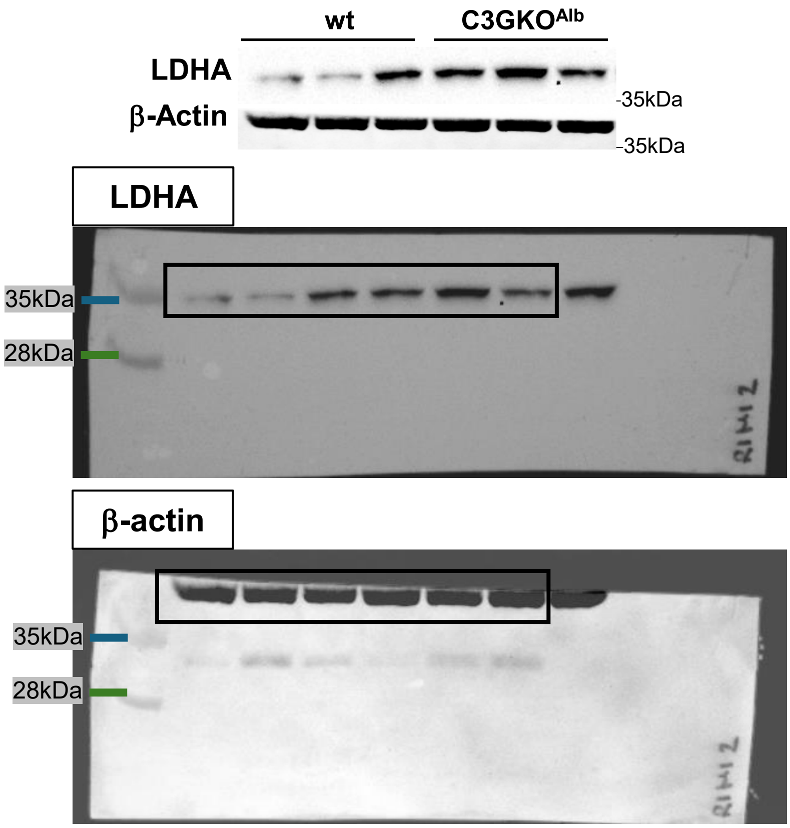
wt
C3GKO^Alb
LDHA
β-Actin
-35kDa
-35kDa
LDHA
35kDa
28kDa
β-actin
35kDa
28kDa

Figure 5A

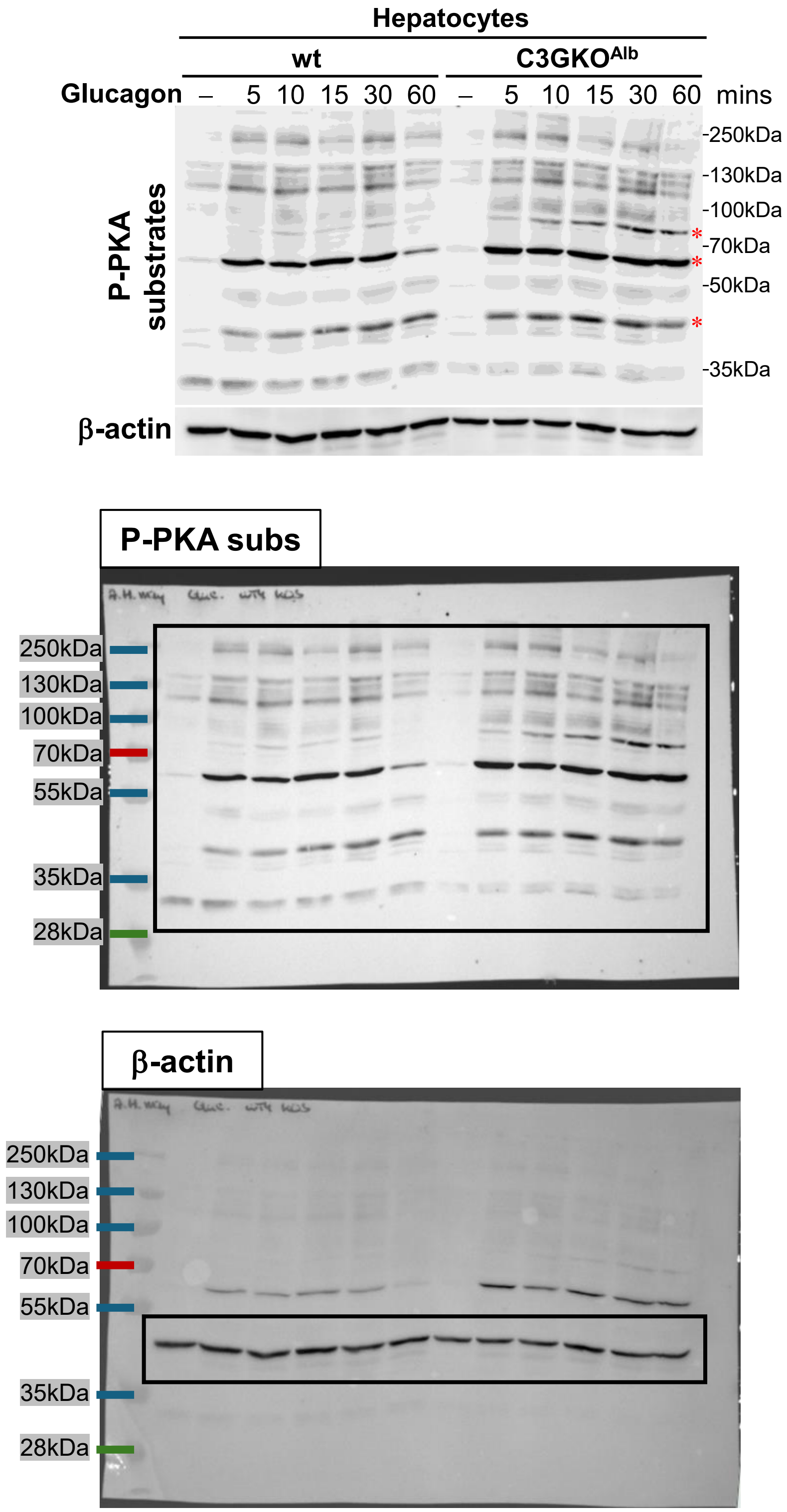
Hepatocytes
wt
C3GKO^Alb
Glucagon – 5 10 15 30 60 – 5 10 15 30 60 mins
P-PKA substrates
β-actin
250kDa
130kDa
100kDa
70kDa
50kDa
35kDa
P-PKA subs
250kDa
130kDa
100kDa
70kDa
55kDa
35kDa
28kDa
β-actin
250kDa
130kDa
100kDa
70kDa
55kDa
35kDa
28kDa

Figure 5B

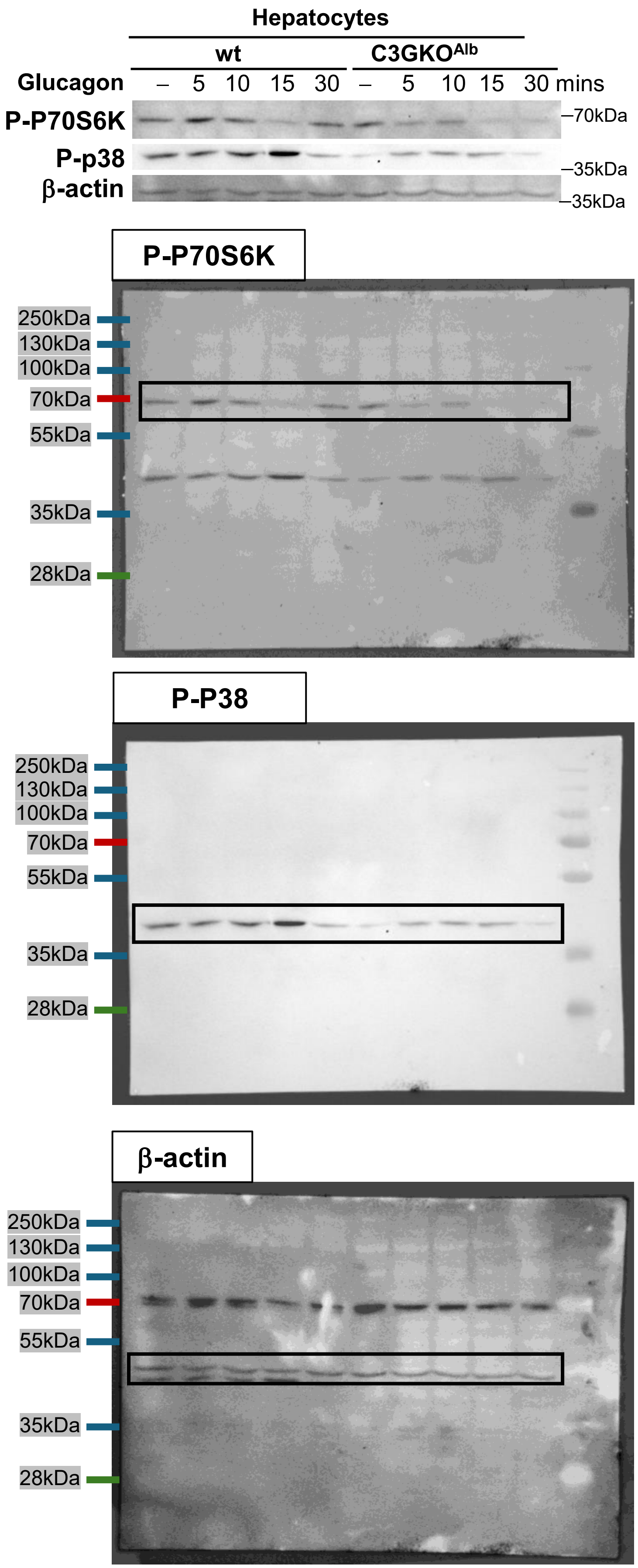

Hepatocytes
wt
C3GKOAlb
Glucagon – 5 10 15 30 – 5 10 15 30 mins
P-P70S6K
P-p38
β-actin
70kDa
35kDa
35kDa
P-P70S6K
250kDa
130kDa
100kDa
70kDa
55kDa
35kDa
28kDa
P-P38
250kDa
130kDa
100kDa
70kDa
55kDa
35kDa
28kDa
β-actin
250kDa
130kDa
100kDa
70kDa
55kDa
35kDa
28kDa

Figure 5C

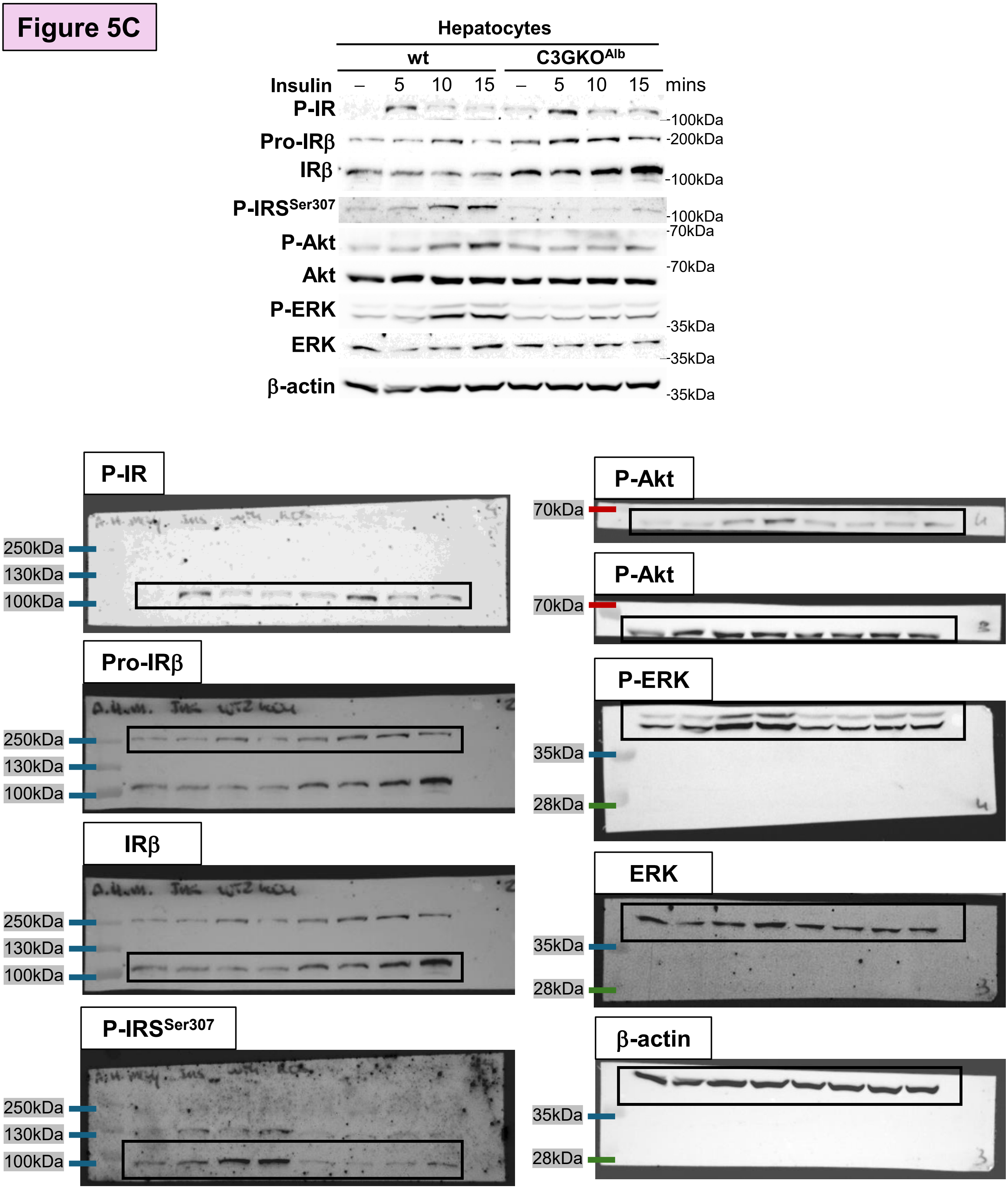

Hepatocytes
wt
C3GKO^Alb
Insulin
–
5
10
15
–
5
10
15
mins
P-IR
Pro-IRβ
IRβ
P-IRS^Ser307
P-Akt
Akt
P-ERK
ERK
β-actin
-100kDa
-200kDa
-100kDa
-100kDa
-70kDa
-70kDa
-35kDa
-35kDa
-35kDa
P-IR
Pro-IRβ
IRβ
P-IRS^Ser307
250kDa
130kDa
100kDa
P-Akt
P-Akt
P-ERK
ERK
β-actin
70kDa
35kDa
28kDa

Figure 6B

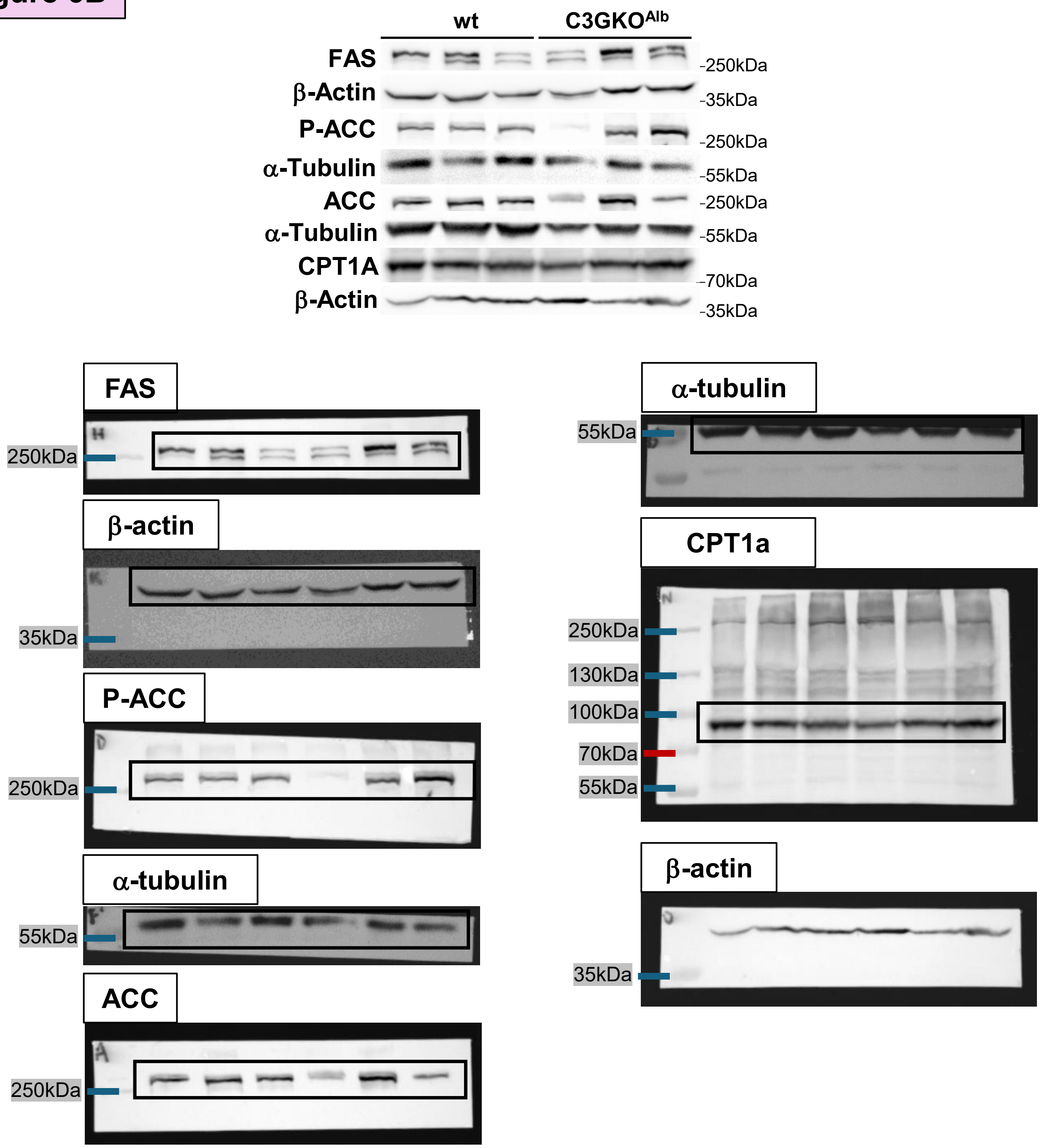

wt
C3GKOAlb
FAS
β-Actin
P-ACC
α-Tubulin
ACC
α-Tubulin
CPT1A
β-Actin
-250kDa
-35kDa
-250kDa
-55kDa
-250kDa
-55kDa
-70kDa
-35kDa
FAS
250kDa
β-actin
35kDa
P-ACC
250kDa
α-tubulin
55kDa
ACC
250kDa
α-tubulin
55kDa
CPT1a
250kDa
130kDa
100kDa
70kDa
55kDa
β-actin
35kDa

Figure 7E

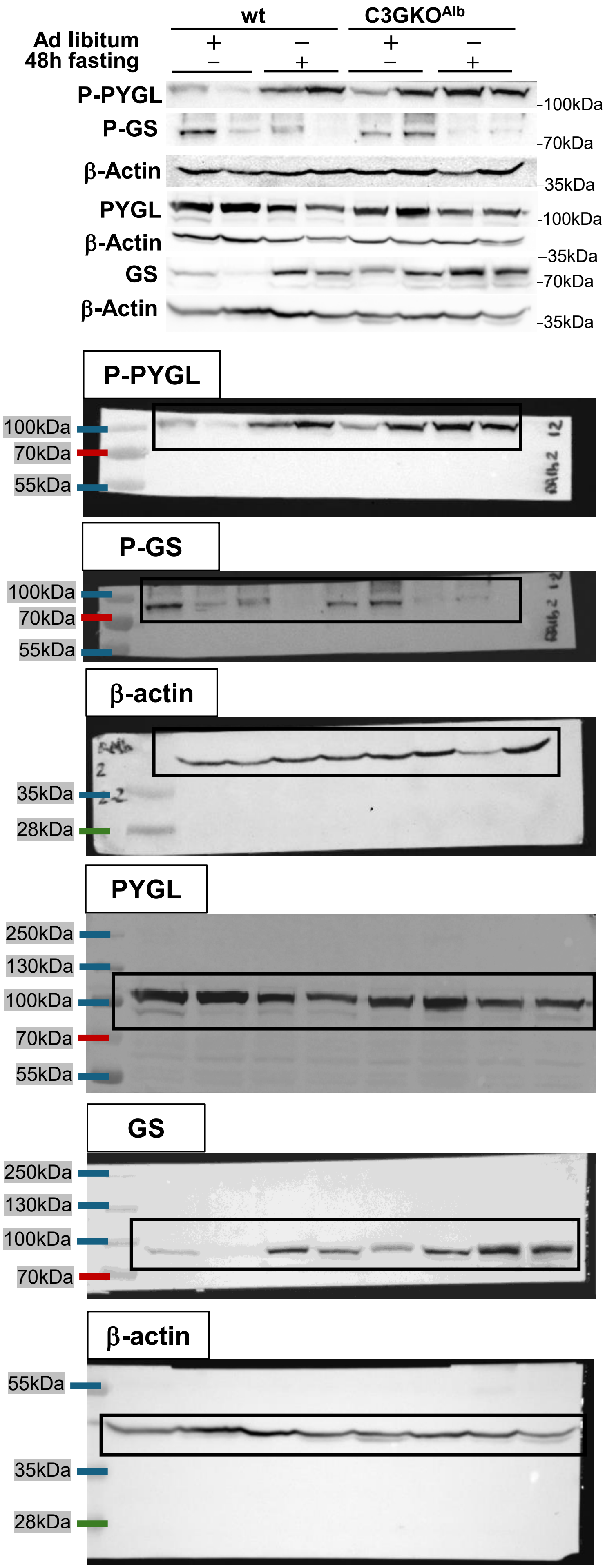

wt
C3GKO^{Alb}
Ad libitum
48h fasting
P-PYGL
P-GS
β-Actin
PYGL
β-Actin
GS
β-Actin
100kDa
70kDa
35kDa
P-PYGL
P-GS
β-actin
PYGL
GS
β-actin
250kDa
130kDa
55kDa
28kDa

Figure 8B

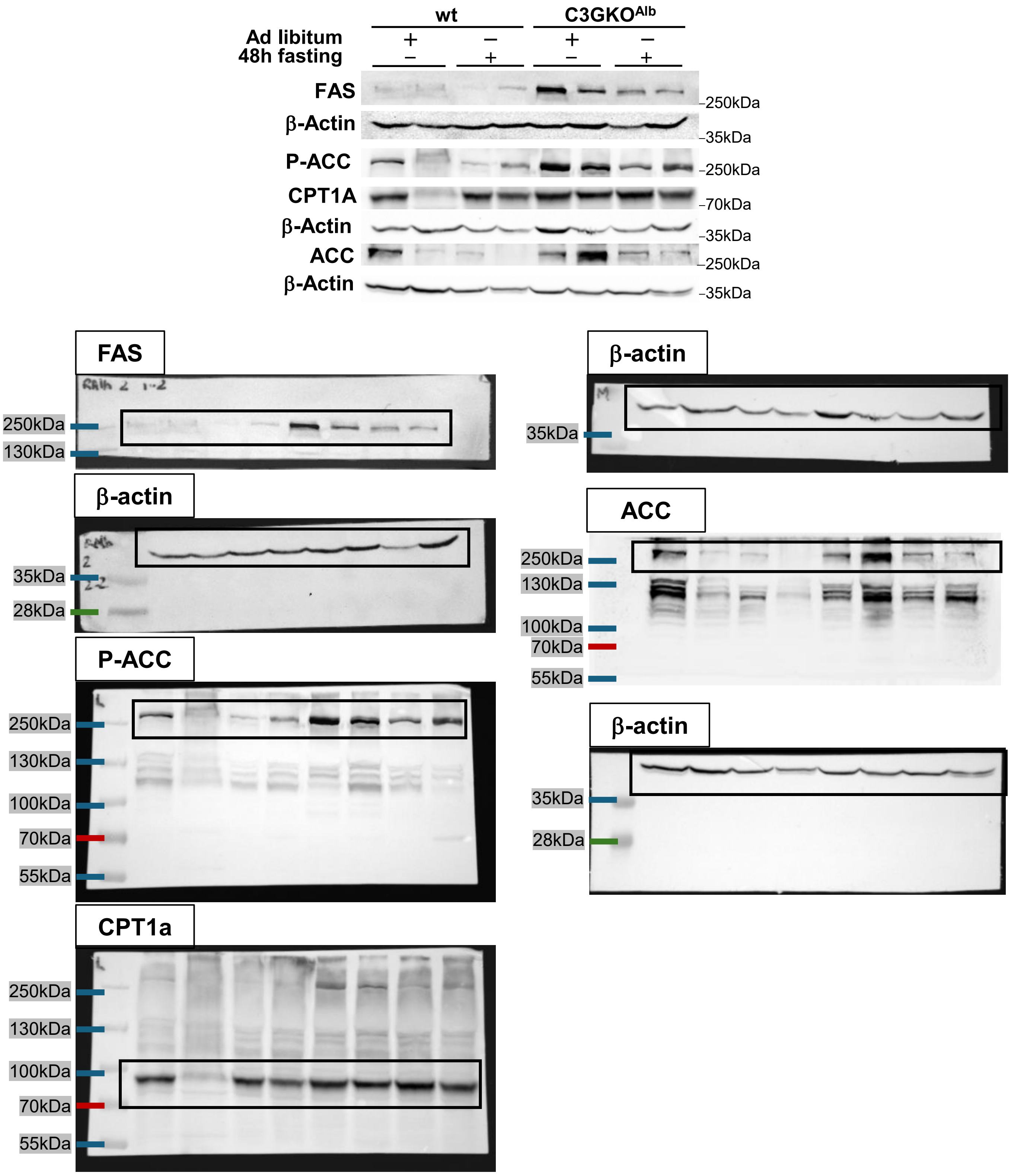
wt
C3GKO^Alb
Ad libitum
48h fasting
FAS
β-Actin
P-ACC
CPT1A
β-Actin
ACC
β-Actin
250kDa
35kDa
250kDa
70kDa
35kDa
250kDa
35kDa
FAS
250kDa
130kDa
β-actin
35kDa
28kDa
P-ACC
250kDa
130kDa
100kDa
70kDa
55kDa
CPT1a
250kDa
130kDa
100kDa
70kDa
55kDa
β-actin
35kDa
ACC
250kDa
130kDa
100kDa
70kDa
55kDa
β-actin
35kDa
28kDa

Supplementary figure 2A

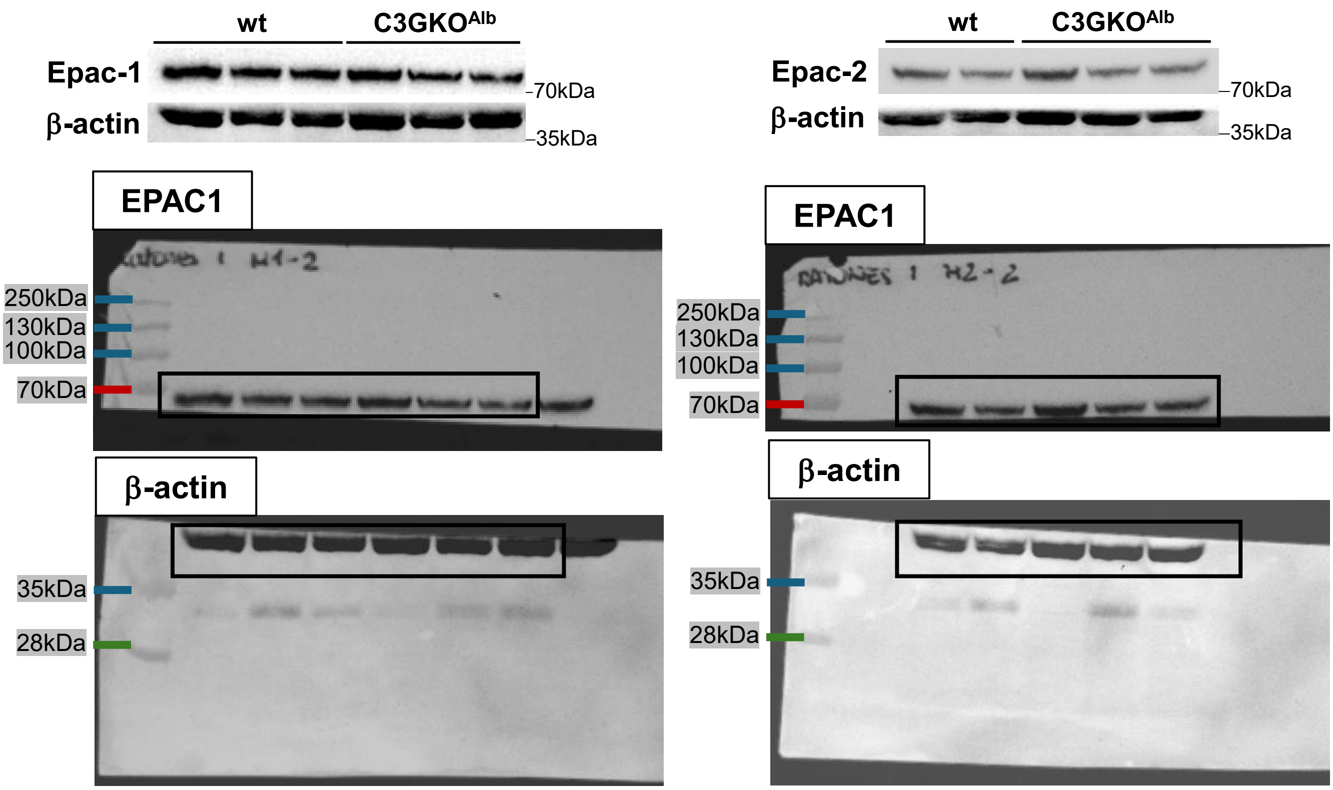

Supplementary figure 2B

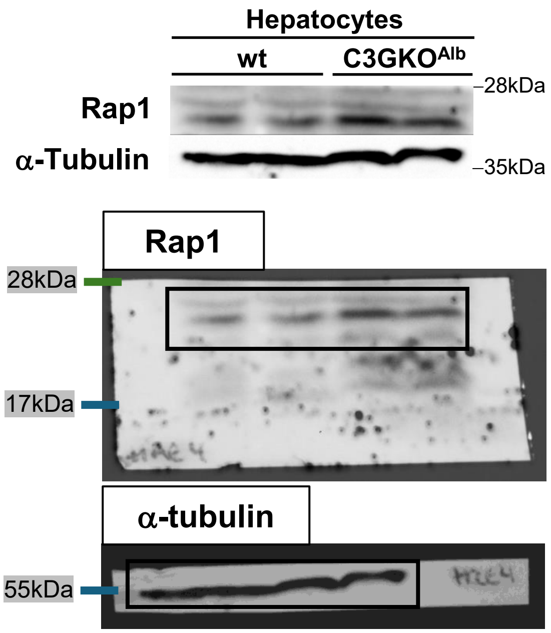

Supplementary figure 5D

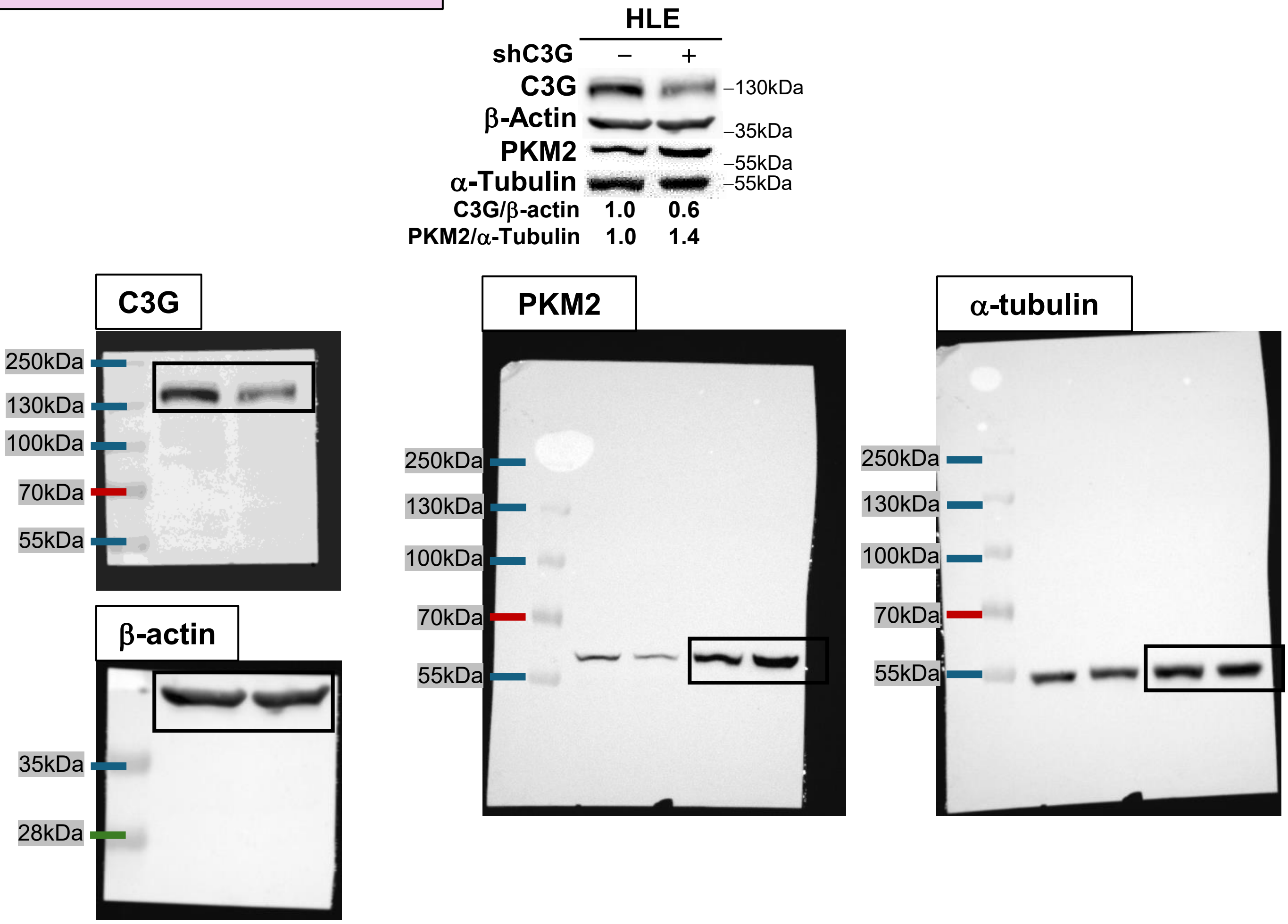

Supplementary figure 5E

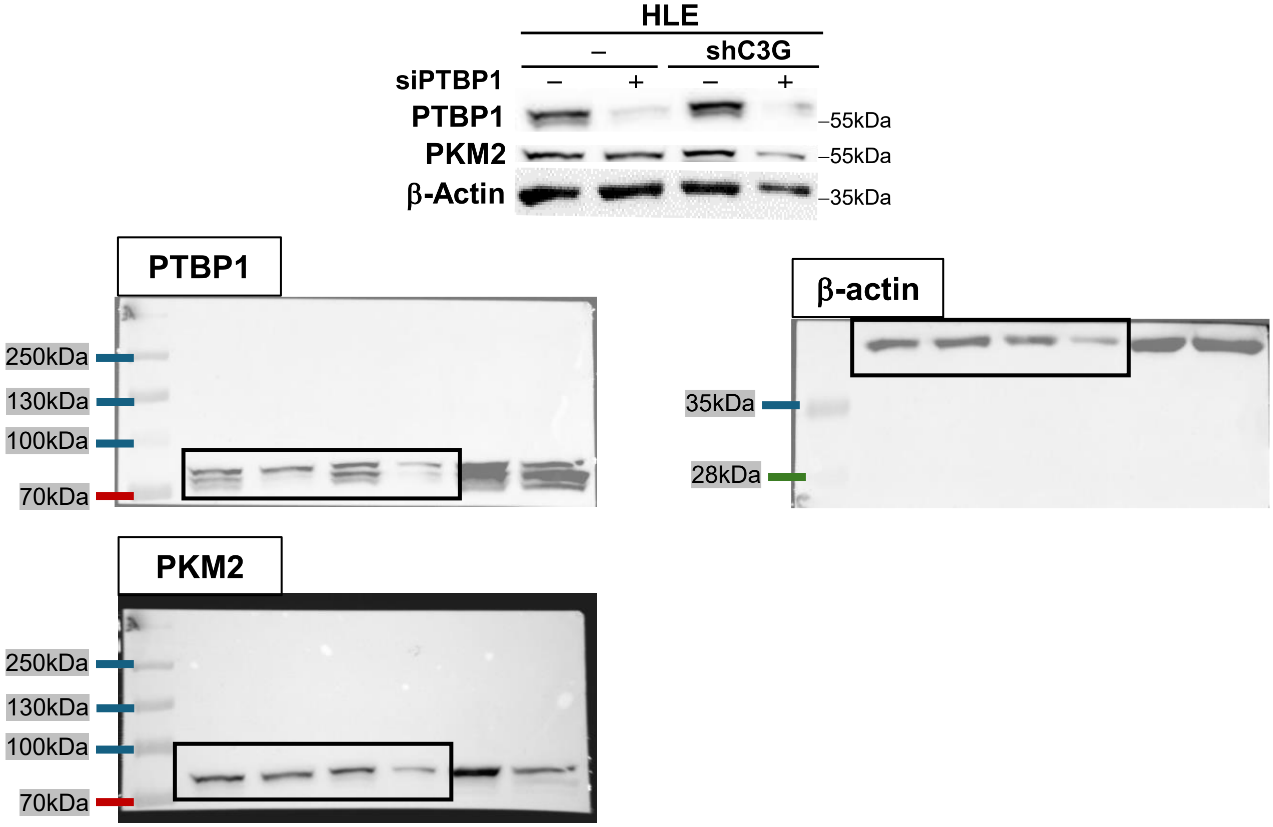

Supplementary figure 6C

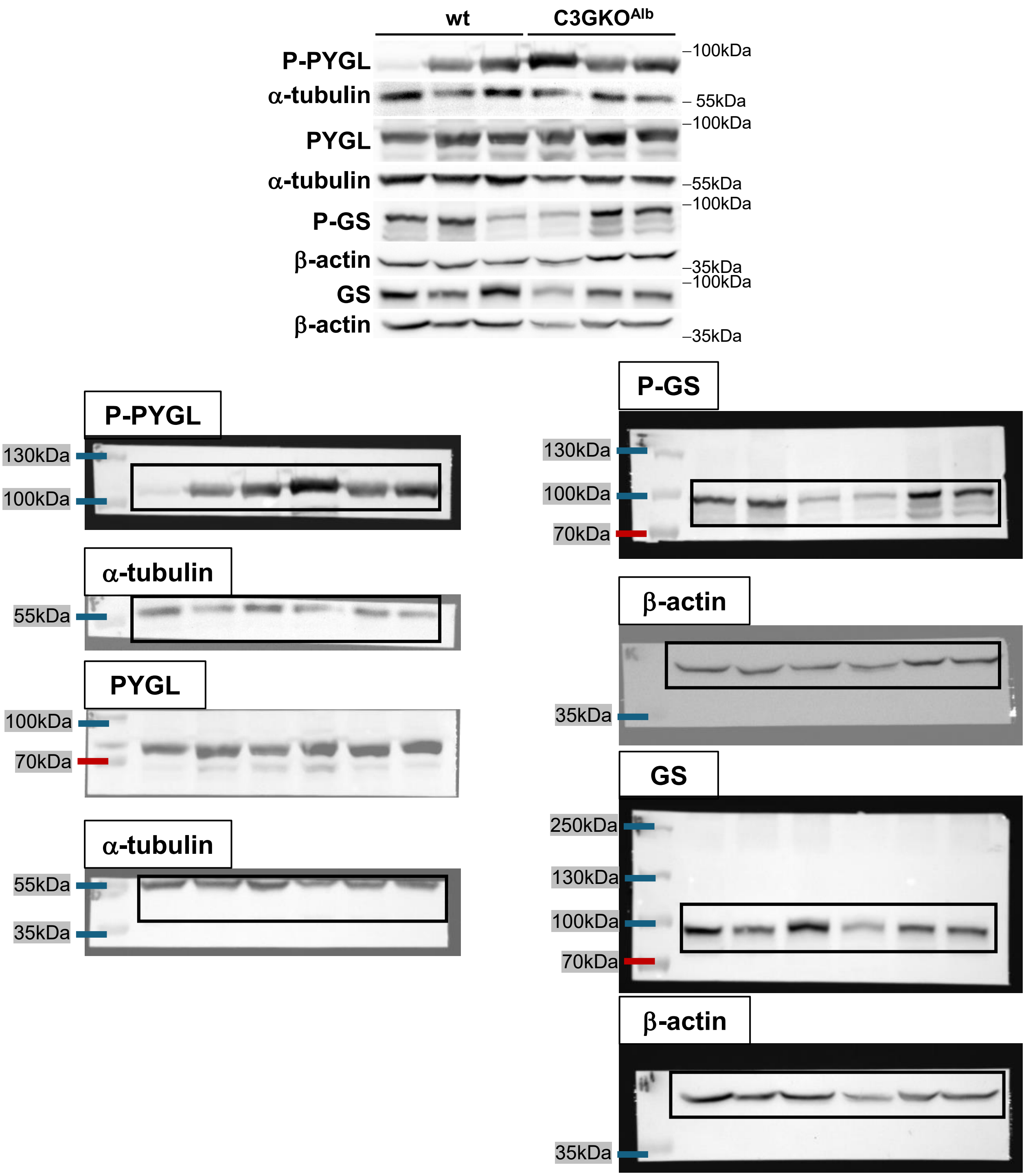

Supplementary figure 8B

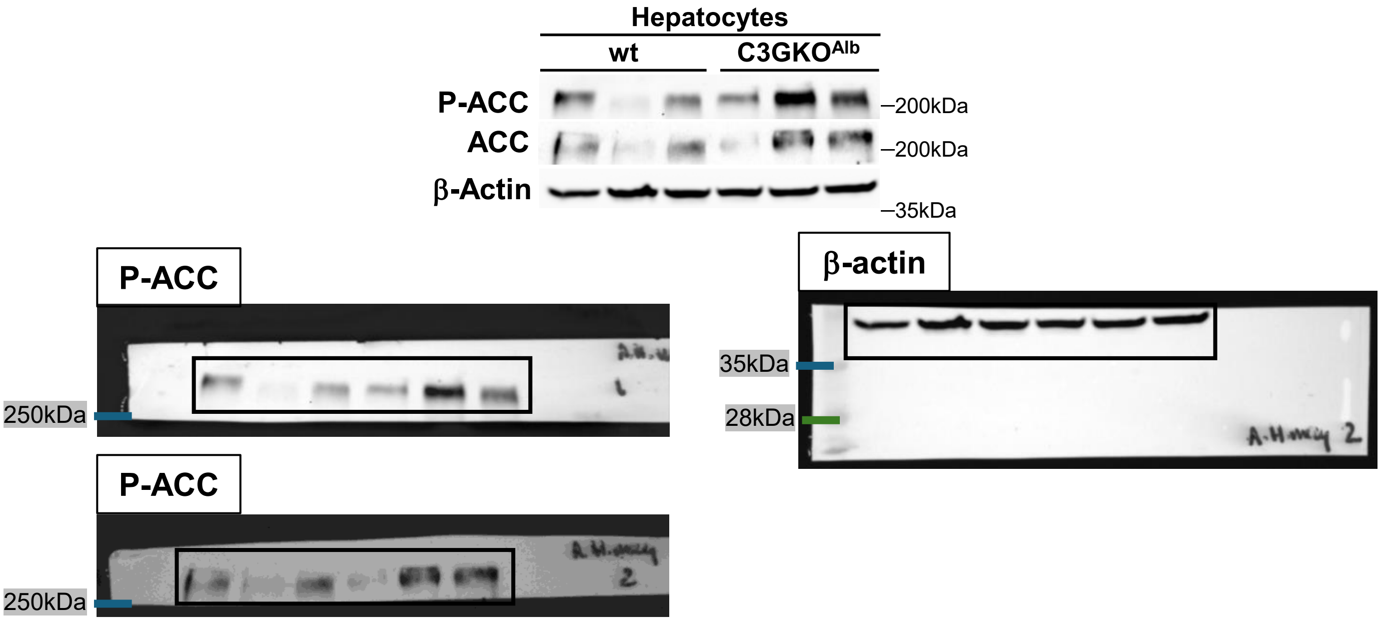

Supplementary figure 8C

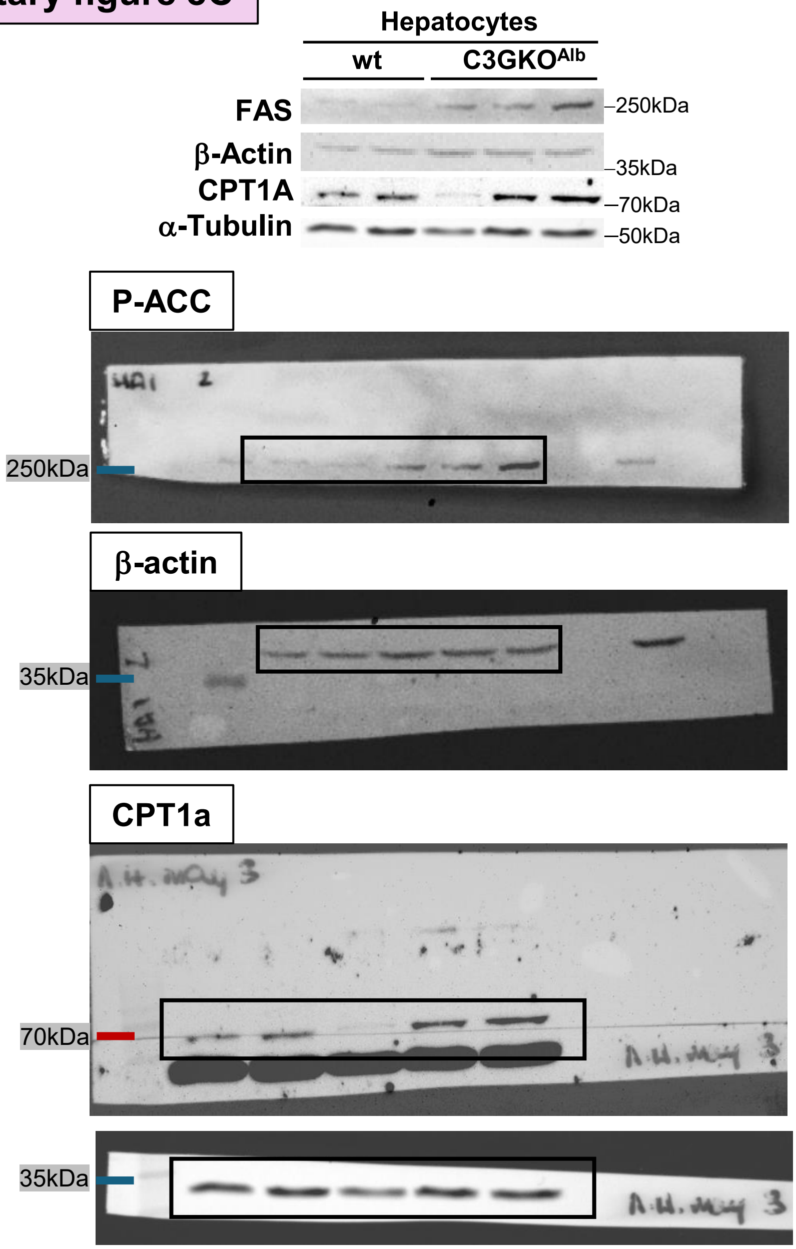

Supplement: Supplementary file 2 — Uncropped western-blots [file 41419_2025_8031_MOESM2_ESM.pdf]
